# Genomic patterns of pathogen evolution revealed by comparison of *Burkholderia pseudomallei*, the causative agent of melioidosis, to avirulent *Burkholderia thailandensis*

**DOI:** 10.1186/1471-2180-6-46

**Published:** 2006-05-26

**Authors:** Yiting Yu, H Stanley Kim, Hui Hoon Chua, Chi Ho Lin, Siew Hoon Sim, Daoxun Lin, Alan Derr, Reinhard Engels, David DeShazer, Bruce Birren, William C Nierman, Patrick Tan

**Affiliations:** 1 Genome Institute of Singapore, Singapore 138672, Republic of Singapore; 2 National Cancer Centre, Singapore 169610, Republic of Singapore; 3 The Institute for Genomic Research, Rockville, MD 20850, USA; 4 Defense Medical and Environmental Research Institute (DMERI), DSO National Laboratories, Singapore 117510, Republic of Singapore; 5 The Broad Institute, Cambridge, MA 02141, USA; 6 Bacteriology Division, US Army Medical Research Institute of Infectious Diseases (USAMRIID), Fort Detrick, MD 21702, USA

## Abstract

**Background:**

The Gram-negative bacterium *Burkholderia pseudomallei *(Bp) is the causative agent of the human disease melioidosis. To understand the evolutionary mechanisms contributing to Bp virulence, we performed a comparative genomic analysis of Bp K96243 and *B. thailandensis *(Bt) E264, a closely related but avirulent relative.

**Results:**

We found the Bp and Bt genomes to be broadly similar, comprising two highly syntenic chromosomes with comparable numbers of coding regions (CDs), protein family distributions, and horizontally acquired genomic islands, which we experimentally validated to be differentially present in multiple Bt isolates. By examining species-specific genomic regions, we derived molecular explanations for previously-known metabolic differences, discovered potentially new ones, and found that the acquisition of a capsular polysaccharide gene cluster in Bp, a key virulence component, is likely to have occurred non-randomly via replacement of an ancestral polysaccharide cluster. Virulence related genes, in particular members of the Type III secretion needle complex, were collectively more divergent between Bp and Bt compared to the rest of the genome, possibly contributing towards the ability of Bp to infect mammalian hosts. An analysis of pseudogenes between the two species revealed that protein inactivation events were significantly biased towards membrane-associated proteins in Bt and transcription factors in Bp.

**Conclusion:**

Our results suggest that a limited number of horizontal-acquisition events, coupled with the fine-scale functional modulation of existing proteins, are likely to be the major drivers underlying Bp virulence. The extensive genomic similarity between Bp and Bt suggests that, in some cases, Bt could be used as a possible model system for studying certain aspects of Bp behavior.

## Background

Identifying the molecular mechanisms and pathways responsible for generating and regulating pathogen virulence is a key challenge of infectious diseases research. Besides increasing our basic understanding of pathogen behavior, such information is also essential for many clinically-relevant areas including the acquisition of drug resistance, vaccine design, and the emergence of new diseases [1,2]. With the increasing availability of complete genome sequence data from multiple microbial pathogens, comparative genomics has recently emerged as a powerful tool to understand the basic molecular properties of pathogens. A particularly useful analysis in this regard has been to compare the genomes of a virulent species to a closely related but avirulent family member. Such studies have revealed the fundamental importance of horizontal acquisition, gene mutation, genome rearrangements, and bacteriophage mediated recombination in the development of virulence [3-5]. However, of the >60 microbial pathogens sequenced to date, less than a quarter (<15) have been compared in this manner. To achieve a broad understanding of the general mechanisms governing the evolution of pathogens, it is likely that such comparative analysis will be required for several more pathogenic species.

The Gram-negative pathogen *B. pseudomallei *(Bp) is the causative agent of melioidosis, a serious, often fatal disease of both humans and animals [6,7]. Endemic to many parts of South East Asia and Northern Australia, Bp is considered a major tropical pathogen [8] and Category B biowarfare agent [9]. In many countries, Bp can only be experimentally manipulated under biosafety level 3 (BSL3) conditions (ABSA, USA). In contrast to Bp, the related species *B. thailandensis *(Bt) is nonpathogenic for humans and animals although it displays several phenotypic characteristics similar to Bp. Indeed, by routine diagnostic tests, it is often difficult to distinguish the two [10]. Like Bp, Bt is also a soil saprophyte, and until its classification as a distinct species in 1998 was considered to represent a subtype of Bp [11,12]. Recently, the genome sequence of one Bt strain, E264, was reported and compared to Bp and *B. mallei*, another related species [13]. However, in that work, most of the comparative analysis was confined to a set of 716 genes transcriptionally regulated in *B. mallei *upon mouse infection, representing less than 15% of all genes in Bp. The identification of Bt-specific genetic elements was also not addressed in that report, which might also prove important for understanding virulence, as recent reports have suggested that gene loss can also contribute to pathogenesis [14]. To definitely address the roles of horizontal transfer, gene mutation, and transcriptional plasticity on the development of Bp virulence, a comprehensive genome-wide comparison of Bp and Bt is required.

In this work, we report the draft genome sequence of Bt strain ATCC700388, corresponding to the same strain as Bt E264. By comparing the ATCC700388 draft sequence against the finished Bt E264 genome, we corrected certain sequencing errors in the reported Bt E264 genome, and compared the refined Bt strain E264 sequence to the genome of Bp K96243 [15]. We find that Bp and Bt are broadly similar at many genomic levels – for example, both species display highly conserved genomic synteny, and appear to share an extensive repertoire of genes involved in core metabolism, accessory pathways, structure-based superfamilies and bacterial virulence factors. Despite these similarities, our analysis also revealed that in comparison to the rest of the genome, virulence-related genes in Bp appear to have undergone accelerated change, perhaps to better adapt to the challenge of infecting and surviving in a human or animal host. We also defined a series of key large-scale differences between Bp and Bt contributing to novel metabolic differences between the two species, and others that may be critically required for virulence. Our results raise several testable hypotheses regarding key virulence components in Bp, and enhance our general understanding of pathogen evolution. Finally, the broad similarities between Bp and Bt also raise the possibility that Bt could prove useful as a potential model organism to study certain aspects of Bp biology.

## Results

### Bt is closely related to Bp by 16S phylogeny

The *Burkholderia *genus is a large bacterial family containing >30 distinct species [16]. We estimated approximate divergence times between Bt and other species in the *Burkholderia *genus by comparing on a phylogenetic basis 16S rRNA gene sequences from four *Burkholderia *species and three outgroup species (*Pseudomonas aeruginosa *LMG1242T, *Escherichia coli *K12 and *Salmonella typhimurium *LT2). From this analysis, we found that Bt occupies a branch in the 16S rRNA phylogenetic tree that is highly related, but not identical, to *B. pseudomallei *(Bp) and *B. mallei *(supplementary figure one in Additional file 1). The close phylogenetic similarity between Bp and *B. mallei *is expected given that recent genome analysis suggests that *B. mallei *is in fact a derivative or clone of Bp [17]. A calibration of the phylogenetic tree against the *E. coli *and *S. typhimurium *divergence, estimated at 140 million years (Mya) [18], suggests that Bt diverged from Bp and *B. mallei *approximately 47 Mya ago. Similar time-frames were also obtained using *groEL*, another highly conserved housekeeping gene (Y. Yu, data not shown). This analysis suggests that although Bt is avirulent, it is likely to be highly evolutionarily related to virulent Bp, and a good candidate for comparative genomic analaysis. Notably, the Bp/Bt separation times are comparable to that used in other studies comparing pathogenic and non-pathogenic species [19,20].

### Genome sequencing of Bt ATCC700388 and comparison to Bt E264

Genomic DNA from Bt ATCC700388 was sequenced by a shotgun approach to 7x genome coverage, and the computer program ARACHNE was then employed to assemble the shotgun reads into contigs and scaffolds. The median contig length was 40 Kb (86 contigs in total), and 3 scaffolds could be assembled from the contigs, comprising 3.4, 2.9, and 0.35 Mb respectively. A subsequent comparison of these scaffolds to the finished Bt E264 sequence allowed the 1^st ^and 3^rd ^scaffold to be collapsed into a single assembly (Chromosome 1). The amount of genome sequence contained in contigs (sum of contig lengths) was 6.66 Mb (Chr 1 – 3.78 Mb, Chr 2 – 2.88 Mb), compared with a total genome length (sum of scaffold lengths, including estimated gaps between contigs) of 6.7 Mb (Chr 1 – 3.80 Mb, Chr 2 – 2.9 Mb). Thus, approximately 99% of the Bt ATCC700388 genome is contained in contigs, with 44 sequence gaps (29 in Chr 1, 15 in Chr 2), of which 15 gaps are larger than 500 bp (largest gap size being 9995 bp).

Although Bt ATCC700388 and Bt E264 represent the same bacterial strain, the specific isolates chosen for sequencing were stored at independent centers (DMERI and USAMRIID), and Bt ATCC700388 exhibits a slightly reduced growth rate compared to Bt E264 (Additional file 2). An analysis of the E264 and ATCC700388 genome sequences revealed that they were near identical except for : I) a chromosomal inversion of 2 Mbp on Chr 1, from position 12442442 (BTH_I1099) to 3328461 (BTH_I2895) based on the E264 genome sequence, II) 4 genes that are absent in ATCC70388, and III) 80 genes with putative protein sequence-altering DNA polymorphisms between the two strains (a full list of these genes is presented in Additional file 3). An initial resequencing analysis of genes in category III) revealed that several of the putative DNA 'polymorphisms' were sequencing artifacts, and thus an extensive analysis of genes in this category was not pursued further (Additional file 3). Of the four ATCC70388 absent genes, two genes (Bth_I1484 and Bth_I1485) were experimentally validated by PCR (Chua HH, data not shown) and encode components of a Type II Oligo-polysaccharide (OPS) synthesis gene cluster [21]. It is possible that the lack of these genes in ATCC700388 may contribute towards the observed growth differences by affecting Bt cell wall and membrane biogenesis. Although this hypothesis is supported by studies of the Bth_I1484 homolog in *B. subtilis *(*TagO*, 22), we emphasize that it is still highly speculative and needs to be further investigated. As the genomes of both strains (ATCC700388 and E264) were largely similar, we decided to adopt the finished E264 genome, after incorporating corrections for a few sequencing errors, as a reference Bt genome to compare against Bp.

### Genome features of Bt

**Figure 1:**
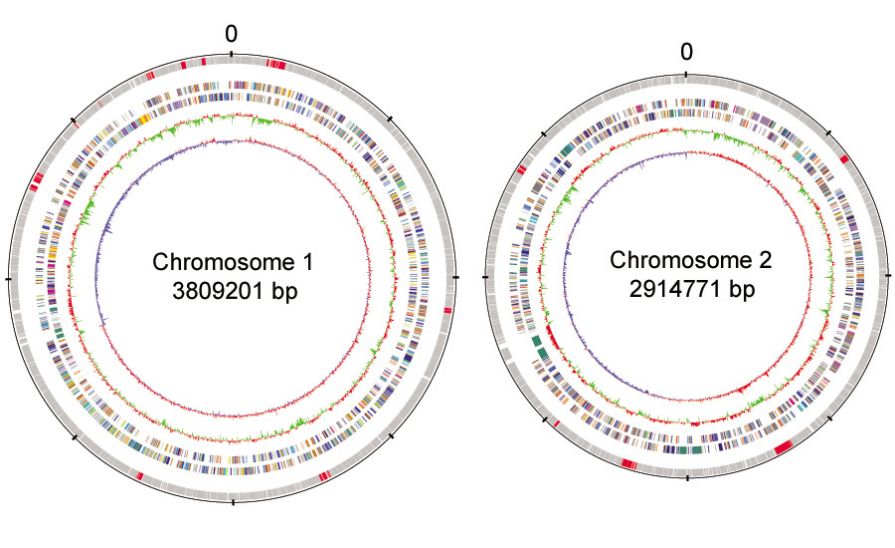
Schematic circular diagrams of the large and small chromosomes of the *B. thailandensis *genome. From outside to inside: scale; annotated CDSs, GIs represented by red; *B. thailandensis *COG categories (two circles), mean centered GC% content plot (red-high GC%, green-low GC%); mean centered (G-C)/(G+C) deviation plot (red-above mean, blue-below mean). Color coding for COG functions: gold, translation; orange, replication and transcription; yellow, nuclear structure; pink, defense mechanisms; tomato, signal transduction mechanisms; peachpuff, cell envelope biogenesis, outer membrane; purple, cell motility and secretion; red, cytoskeleton; green, extracellular structures; royalblue, energy metabolim; blue, central metabolism; aquamarine, secondary metabolism; gray, function unknown.

Similar to Bp, the Bt genome comprises two circular chromosomes of 3.8 and 2.9 Megabases (Figure 1). With a total of 5,645 predicted protein-encoding ORFs (3282 from Chromosome 1 and 2363 from Chromosome 2) (Table 1), the Bt genome ranks in the top twelve largest bacterial genomes amongst the 200-odd bacterial genomes sequenced at the time of writing this report (supplementary figure two in Additional file 1). A striking architectural feature of the Bt genome is the presence of at least fifteen regions exhibiting either atypical GC content or stretches of bacteriophage related genes and phage-like integrases (supplementary table one in Additional file 1). Collectively encompassing 4–5% of the entire genome, we refer to these regions as Bt-genomic islands (Bt-GIs), in keeping with similar regions found in Bp [15]. The Bt-GIs are not found in Bp and *B. mallei*, consistent with their being acquired by Bt subsequent to the Bp-Bt divergence. Supporting a model of recent acquisition, it has been found that some of these regions (eg. Bt-GI12) may still harbor functionally active bacteriophages [23]. Interestingly, of the ten Bt-GIs on Chromosome 1 and five on Chromosome 2, three Bt-GIs (Bt-GI 8, Bt-GI 9, and Bt-GI 10) occur in the same relative genomic location as GIs in the Bp genome. The presence of different horizontally acquired sequences in the same genomic location in related species suggests that these locations may represent genomic hot spots or landmarks for the acquisition of horizontally acquired sequences.

**Figure 2:**
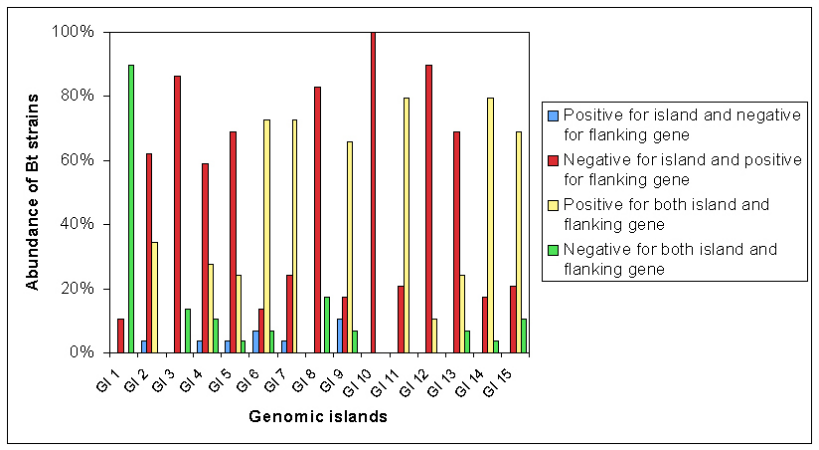
Prevalence of Bt-GIs in natural isolates of Bt. Distribution of Bt-GIs in Bt isolates (n = 29) as determined by PCR. For each GI, two genes were tested: the target genes that locate in the GIs and the flanking genes that are immediately abutting each putative island. This figure depicts the four possibilities for each genomic island: Positive for island and negative for flanking gene (blue), Negative for island and positive for flanking gene (red), Positive for both island and flanking gene (yellow), Negative for both island and flanking gene (green).

To experimentally show that the Bt-GIs are likely to represent regions of lateral transfer between different Bt strains, we then performed a series of PCR experiments to confirm the presence or absence of the fifteen Bt-GIs in 29 natural isolates of Bt, obtained from independent soil locations in either Northeast (n = 21) or Central Thailand (n = 8, 9 if E264 is also considered). We found that the distribution of Bt-GIs was indeed highly variable between different Bt strains (Figure 2). Specifically, while Bt-GIs 1, 3, 8 and 10 appear to be unique to Bt E264, GIs 2, 4, 5, 12, 13 were detected in 10–50% of strains and the rest of the GIs were detected in 50–80% of strains. Interestingly, GI 12 was present in 4 out of 9 strains isolated from Central Thailand (including E264), but absent from all Northeast Thailand strains. These results indicate that the Bt genome, like Bp, indeed contains a substantial fraction of genomic material that is highly variable in different strains. It is thus possible that genes on these islands may contribute to the presence of different strain-specific phenotypes.

### Genomic synteny and large-scale differences between the Bp and Bt genomes

**Figure 3:**
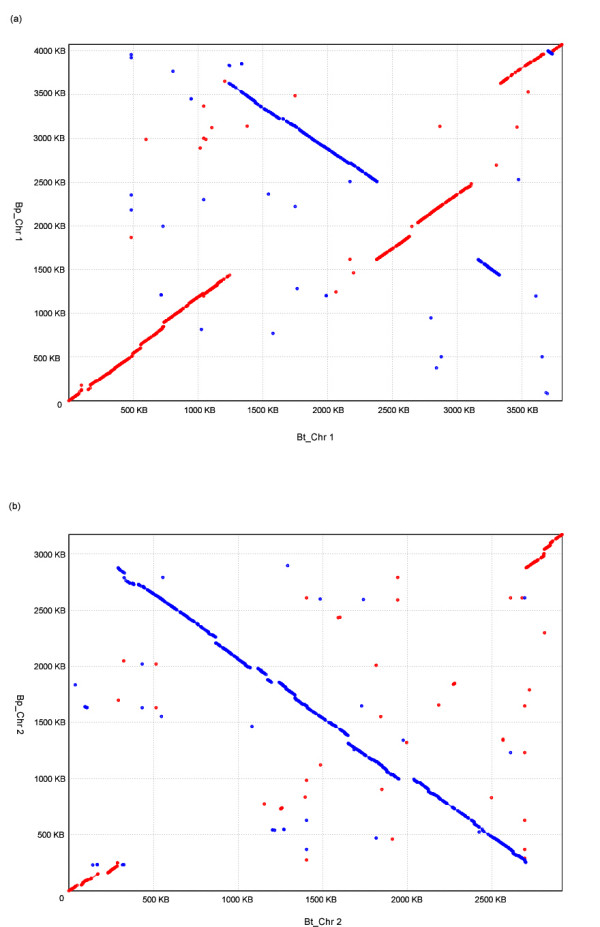
Genomic Synteny of theBp and Bt genomes. The Bt and Bp genomes are depicted on the x and y axes respectively. (a) Bt chr 1 forward strand versus Bp chr 1 forward strand; (b) Bt chr 2 forward strand versus Bp chr 2 forward strand. Inverted orientations of segments indicate regions of genomic inversion.

To assess large-scale genomic similarities between Bp and Bt, we aligned the two genomes and found that both chromosomes were highly syntenic between the two species (Figure 3). Specifically, we identified only four large-scale inversions involving genomic regions greater than 10 Kb; three in Chromosome 1 and one in Chromosome 2. Six out of the eight breakpoints flanking these inversions contain genes involved in DNA recombination such as transposases, phage integrases and recombinases (supplementary table two in Additional file 1). At this large-scale resolution, we did not observe any evidence for exchange of genetic material across the two chromosomes. The high degree of synteny between Bt and Bp is unexpected, given the fundamental plasticity of bacterial genomes, and because a previous analysis has demonstrated a much higher degree of genome rearrangement events between Bp and *B. mallei*, which is even more closely related [17]. The syntenic conservation of Bp and Bt is also considerably higher than that observed between other virulent and non-virulent bacteria species, despite the latter having smaller genome sizes and similar or shorter divergence times from that of Bt and Bp [5]. This unexpected result raises the possibility that that there may exist certain functional constraints acting as a negative selective forces to preserve the large-scale genomic organization of Bp and Bt.

The acquisition and loss of large-scale genomic material represents a major driving force in bacterial evolution and often plays a critical role in the development of novel microbial phenotypes. These species-specific regions, which include the Bp-GIs and Bt-GIs, could largely be collapsed into a series of discrete genomic stretches, often exceeding 100 kb. We classified these regions into three categories: I) Bp-specific, representing regions present in Bp but absent in Bt; II) Bt-specific, for regions present in Bt but absent in Bp; and III) Bp-Bt-divergent, representing regions of distinct CDS content but which are present in both species at similar genomic locations. We now discuss a few interesting examples from these three categories (see Table 2), and a comprehensive list is provided supplementary table three (see Additional file 1). Notably, we have deliberately excluded the Bp and Bt GIs from this analysis, since they have already been described earlier and in a previous report [15].

#### Metabolism

**Figure 4:**
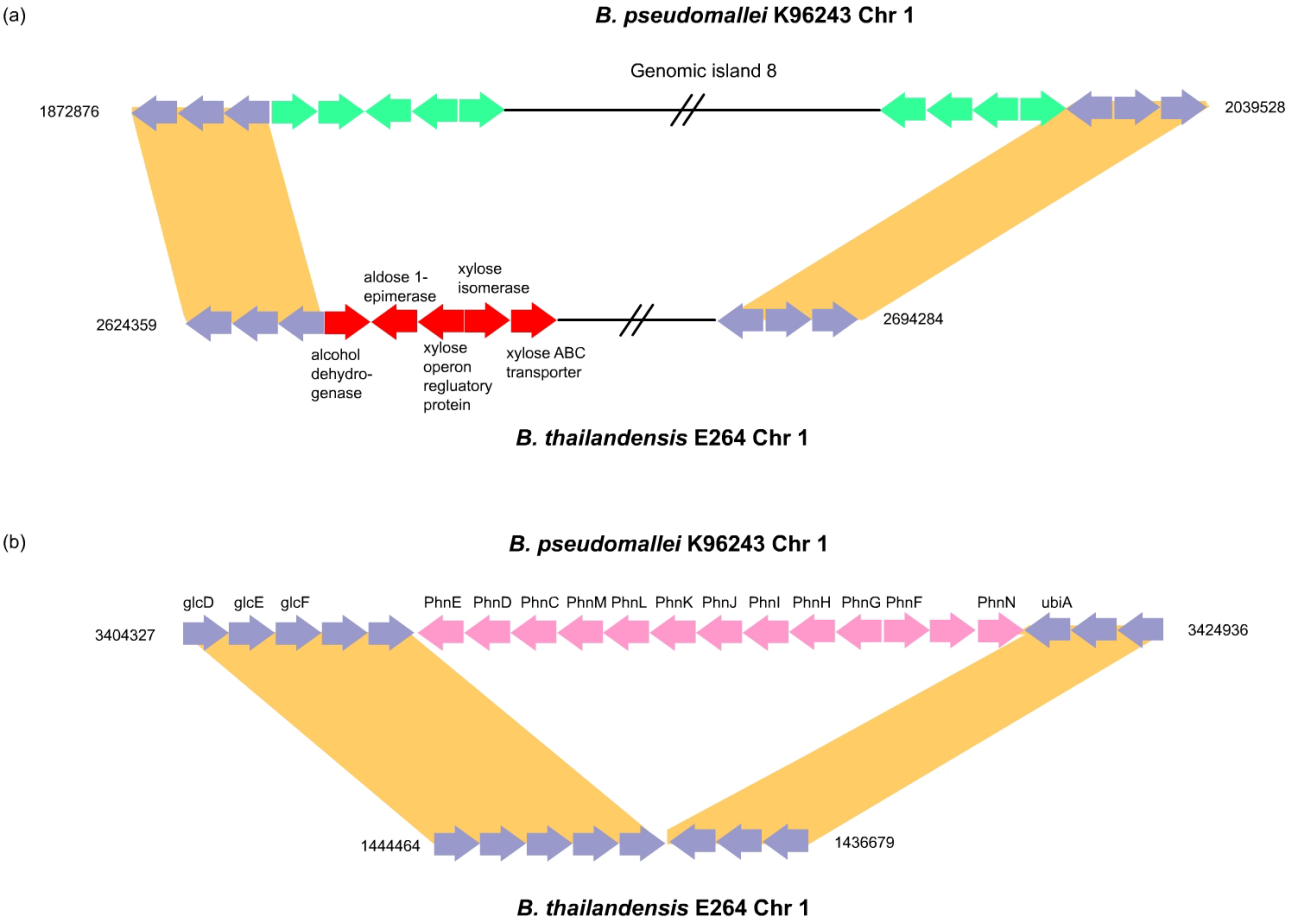
Comparison of xylose operon and phosphonate operons. (a) Insertion of Genomic island 8 (Bp-GI 8) and deletion of xylose operon (red) in Bp. The xylose utilization operon in Bt is absent in Bp, being replaced by Bp-GI 8. (b) A phosphonate gene cluster (pink) is present in Bp while absent in Bt. Genes in purple represent orthologous proteins. See Main Text for details.

Although Bp and Bt occupy similar ecological niches, previous biochemical analysis has identified several phenotypic differences between the species, including the ability of Bt, but not Bp, to assimilate the carbon sources arabinose and xylose [24]. Confirming a previous report [25], we found that Bt contains an eight-gene arabinose assimilation operon (BTH_II1626–1633) on Chromosome 2 that is absent in Bp. In Bp, this region has been replaced by a two protein cluster containing one hypothetical protein and one MarR family regulatory protein. A similar situation is likely to explain the differences in xylose metabolism as well. We found that Bt contains a 64 kb region on Chromosome 1 encoding several genes involved in xylose metabolism, including an aldose epimerase, xylose isomerase, xylose ABC transporter and an operon regulator (BTH_I2336–2339). This region, however, is absent in Bp, which instead carries GI 8 in the same location (Figure 4a). This result suggests an evolutionary model where the horizontal acquisition of GI 8 by Bp may have also resulted in deletion of the xylose gene cluster, demonstrating how horizontal transfer events can often result in simultaneous gene acquisition and loss. We also identified a thirteen-gene phosphonate gene cluster (BPSL2848–2860) on Chromosome 1 in Bp that was absent in Bt, consisting of ATP-binding (*phnC, phnKL, phnN*), phosphonate binding (*phnD*), metabolism (*phnM, phnJ-G*), and transport proteins (*phnE*), and a transcriptional regulator (*phnF*) (Figure 4b). In other species, gene products of the *phn *operon are required for the utilization of phosphonate as a phosphorus source [26], and are also involved in the uptake of phosphite, phosphate, and phosphate esters such as phosphoserine [27]. Since phosphorus availability is essential for many aspects of cell physiology, the lack of this operon in Bt suggests that other mechanisms must exist for acquiring phosphate in Bt. Supporting this hypothesis, the Bt genome contains several genes that could function in alternative pathways for phosphorus acquisition, including alkylphosphonate (BTH_I0257), phosphoglycerate (BTH_I3017–3022), and l-phosphonoalanine (BTH_II1908–1910) gene clusters.

#### Virulence

**Figure 5:**
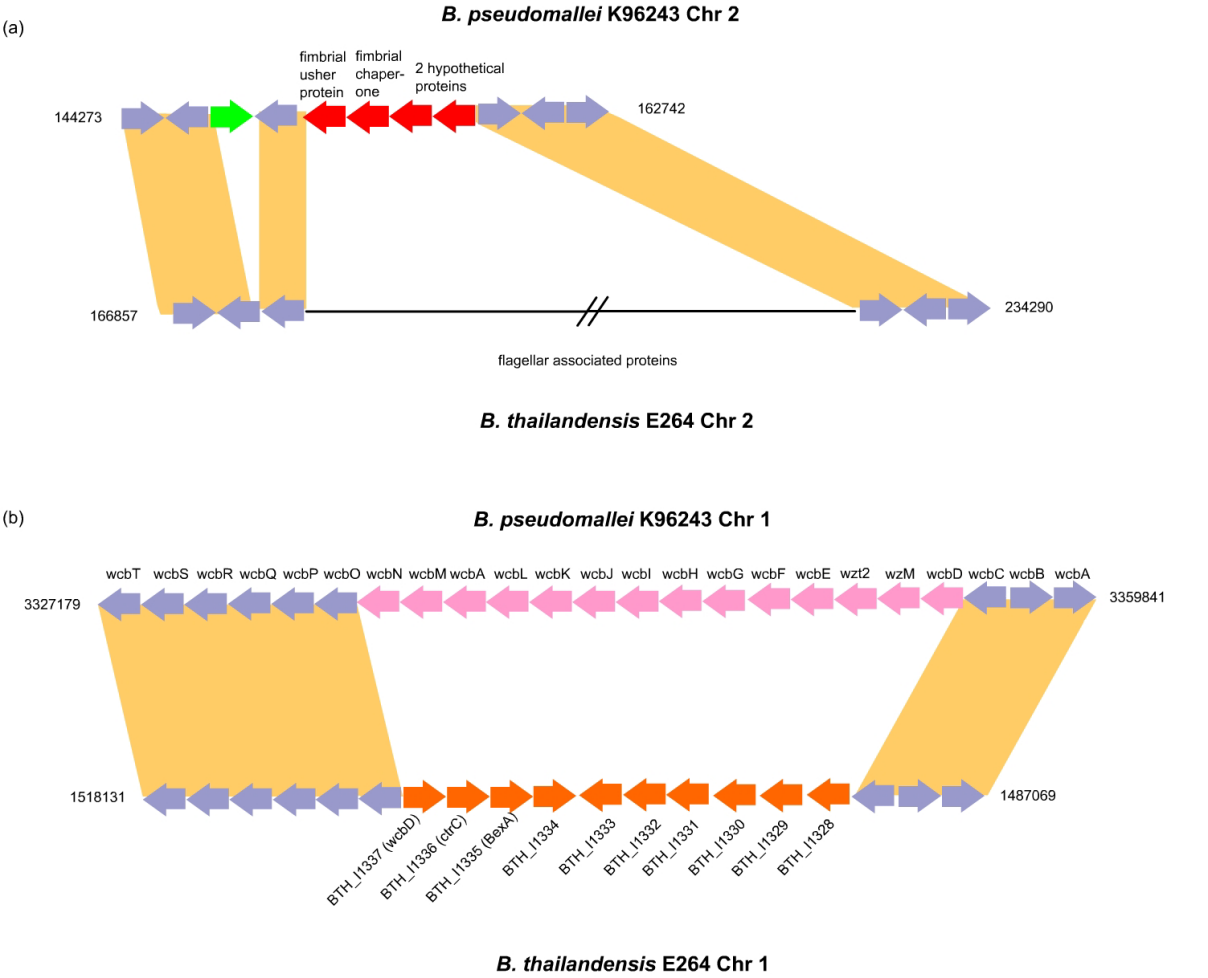
Comparison of fimbriae and capsule biosynthesis regions. (a) Horizontal acquisition of a *Yersinia*-like fimbriae cluster (red) by Bp results in reaplcement of a flagellar biosynthesis cluster in Bt. (b) A capsular polysaccharide biosynthesis cluster (pink) in Bp replaces an ancestral polysaccharide cluster in Bt (orange). See Main Text for details.

We attempted to identify genomic regions that might specifically contribute to the differential virulence of Bp and Bt. Variations in surface component proteins have been shown to contribute to virulence in several pathogenic species. For example, fimbriae are short pilus structures that allow bacteria to adhere to environmental surfaces and host cells, and Bp has been shown to be more efficient than Bt in adhering to and invading host cells [28]. We found that Bp contains twice as many fimbrial gene clusters (six) as Bt. Of the three Bp-specific fimbrial gene clusters (BPSL1626–1629, BPSL1799–1801, BPSS0120–0123), one cluster on Chromosome 2 is of particular interest as it contains a fimbrial usher protein, a fimbrial chaperone and two exported proteins with similarity to fimbriae proteins of *Yersinia *spp, whose family member *Y. pestis *is the causative agent of plague. This cluster appears to have been horizontally acquired by Bp as its GC content significantly deviates from the rest of the Bp genome, and replaces a flagellar operon (BTH_II0143–0197) in the homologous position in the Bt genome (Figure 5a). In addition to fimbriae, another important class of surface-related proteins that could contribute to virulence are surface polysaccharides. It has been previously shown by other molecular techniques that Bp contains a large gene cluster (BPSL2790–2810) involved in the synthesis and export of capsular polysaccharides, a major determinant of virulence, and that this cluster is absent in Bt [29]. We confirmed this finding and further discovered that the specific location of the Bp capsule gene cluster within the Bp genome is likely to be non-random, as it replaces a pre-existing 10-gene cluster in Bt already dedicated towards the metabolism and processing of polysaccharide structures (Figure 5b). Specifically, the original cluster in Bt contains several genes involved in polysaccharide assembly, such as GDP-6-deoxy-D-lyxo-4-hexulose reductase, a GDP-mannose 4, 6-dehydratase, a methyltransferase and four glycosyltransferases, which are all involved in GDP-D-rhamnose biosynthesis in *Pseudomonas aeruginosa *[30]. This result expands our depth of understanding surrounding this important acquisition event, and raises the intriguing possibility that a key event in the pathogenic evolution of Bp was to replace a pre-existing or ancestral polysaccharide coat with an alternative pathogenic variant capable of resisting challenges by the immune system of an infected host. In addition to surface molecules, another broad class of molecules that might contribute to virulence is secondary metabolites with toxin activities [31]. There are four polyketide synthase (PKS) and nonribosomal pepeide synthase (NRPS) clusters involved in the production and regulation of secondary metabolites that are present in Bp but absent in Bt, containing genes encoding putative resistance proteins (BPSS1011), efflux proteins (BPSS1268) and lipopeptide antibiotic proteins (BPSS1631–1634), which could be involved in toxin production. Other differences, including the previously reported absence of a type III secretion system (TTS1) in Bt [32], are listed in the supplementary table three and table four (see Additional file 1).

### The Bp and Bt genome share similar protein family distributions

The SUPERFAMILY hidden Markov model library [33] is a database representing all proteins of known structure derived using Structural Classification of Proteins (SCOP) defined structural folds. We used SUPERFAMILY analysis to classify and compare evolutionarily related groups of domains between the two organisms. We queried the predicted proteomes of Bp and Bt against the SUPERFAMILY HMM library and obtained structural (and hence implied functional) assignments to protein sequences at the superfamily level for both species. This exercise produced 3706 Bt sequences with 721 unique SCOP superfamilies and 3645 Bp sequences with 705 unique SCOP superfamilies. The top 20 structural families assigned to Bp and Bt protein sequences are provided in supplementary table five (see Additional file 1). We found the distribution of different structural family assignments highly similar between Bp and Bt, supporting our basic conclusion that genomes of Bt and Bp are highly conserved. The most populated superfamily in both organisms were P-loop containing nucleoside triphosphate hydrolases (~7.7%), with a local sequence motif ([AG]-x(4)-G-K-[ST]) for ATP/GTP binding. The second and third most distributed superfamilies are winged helix DNA-binding domain (~6%) and NAD(P)-binding Rossmann-fold domains (~5%), which are involved in DNA-binding and NAD(P)-binding respectively.

### Virulence genes in Bp and Bt exhibit increased diversity

Besides the acquisition of large-scale genomic material, alterations in the amino acid composition of protein homologs could also play an important role in the phenotypic differences of Bp and Bt, by altering protein activities and specificities. To perform a systematic analysis of the Bp and Bt proteomes, we identified and compared 4630 orthologous protein pairs (2826 from Chromosome 1 and 1804 from Chromosome 2) between the two genomes, and mapped them to them to their respective metabolic pathways. We found strong conservation between the Bp and Bt proteomes in core metabolic pathways such as amino acid metabolism, cofactor and carrier synthesis, nucleotide and protein biosynthesis, consistent with the ability of Bp and Bt to occupy similar environmental niches [24]. Based upon KEGG annotations, only five out of 1997 genes involved in core metabolic pathways in Bp do not have a clear homolog in Bt, including a putative ATP-binding protein (BPSL2860), a putative acetyltransferase (BPSL1417), a putative RNA 2'-phosphotransferase (BPSL0762) and two ABC transporters (BPSL1824, BPSL2849). This result suggests that the central metabolic machineries utilized by Bp and Bt are likely to be highly similar. Unexpectedly, the Bp and Bt proteomes also appear to share significant similarities in their virulence components as well. Specifically, of 368 known and potential virulence genes in Bp [15], 275 orthologs (71%) are present in Bt at an average similarity of greater than 80% (see supplementary table four in Additional file 1), including two type three secretion systems (TTS2 and TTS3), antibiotic resistance genes, type IV pili-generating proteins, hemolysin-related genes, and several adhesion factors and proteases. Of the remaining 93 Bp-specific virulence-related genes, 20 are located in Bp GIs or regions of atypical GC content, and 73 within the core Bp genome.

**Figure 6:**
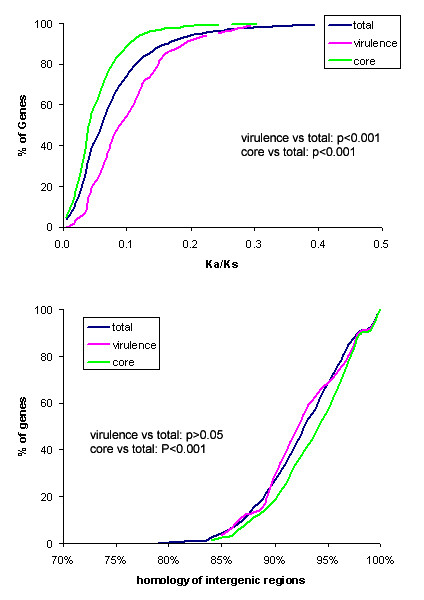
Ka/Ks substitution curves and similarity comparisons of promoter regions. (a) Ka/Ks substitution curves for the orthologous virulence proteins, core metabolism proteins and total proteins. The y axis is the cumulative percentage for the corresponding Ka/Ks value on the x axis. The P values depict the statistical difference between virulence genes or core genes compared to the whole genome (b) Similarity of promoter regions for the orthologous virulence proteins, core metabolism proteins and total proteins. The y axis is the cumulative percentage for the corresponding homologous value on the x axis. The P value depicts the statistical difference between virulence or core genes to the whole genome.

We then investigated if specific components of the Bp and Bt genomes might exhibit significantly greater or lesser levels of diversity, by computing synonymous and non-synonymous nucleotide substitution rates (Ka and Ks) for orthologous protein pairs. The average Ka/Ks ratio for Bp and Bt orthologs was 0.069 (0 – 1.14) for Chromosome 1 and 0.099 (0 – 1.20) for Chromosome 2, where low ratios suggest decreased diversity (p < 0.001 for Chr1 vs Chr2). The significantly lower Ka/Ks ratio for Chromosome 1 is consistent with this chromosome encoding many proteins involved in core biological functions, including energy production and conversion genes, genetic information processing, cell cycle control. Supporting this hypothesis, the Ka/Ks ratio of core metabolism genes (0.05) from both chromosomes was significantly lower than the genome average (p < 0.001). In contrast, the average Ka/Ks ratio of ortholgous virulence genes (0.099) was significantly higher than the genome average (p < 0.001, Figure 6a), suggesting that these virulence-associated genes are likely to exhibit increased diversity between Bp and Bt compared to other genes. Intriguingly, genes associated with the Type III secretion cluster TTS3, including BPSS1547 (*prgH*), BPSS1549 (*mxiI/prgJ*), BPSS1551 and BPSS1552 (*orgA *and *B*), exhibited the highest level of divergence among the virulence related genes between Bp and Bt. In other species, *prgH *and *prgJ *proteins are integral members of the Type III secretion needle complex, which establishes a specific physical interface between the pathogen and host [34]. It is tempting to speculate that subtle functional alterations in these specific TTS3 genes could have contributed towards the ability of Bp to successfully infect human and other animal hosts. In addition to the TTS3 members, four genes of unknown cellular function (one from chromosome 1 and three from chromosome 2) also exhibited Ka/Ks ratios greater than one, suggesting that they might be rapidly evolving. However, no predictable function or identified domains could be assigned to these four genes, thus not allowing speculation on their roles in pathogenesis.

### Functional biases in Bp and Bt pseudogenes

**Figure 7:**
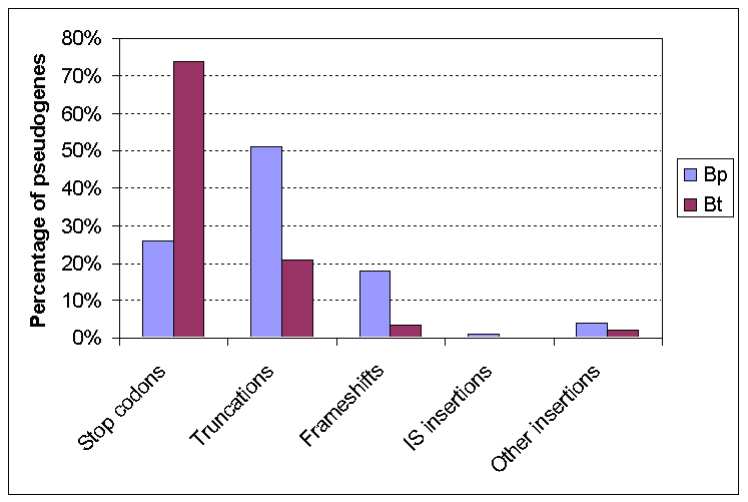
Abundance graph for pseudogenes across different categories of functional inactivation. The x-axis depicts the multiple types of mutations that can cause pseudogene formation in Bp (blue) or Bt (purple).

Besides point differences in amino acid composition, another evolutionary mechanism for generating protein diversity is the introduction of point or frameshift mutations to generate pseudogenes that are subsequently eroded and eliminated by small deletions events [35]. We used the Psi-Phi program to identify pseudogenes in Bp and Bt [36], and found 170 (129 on Chr1 and 41 on Chr2) pseudogenes in Bp and 179 pseudogenes (82 on Chr1 and 97 on Chr2) in Bt. Of the 170 Bp pseudogenes, 44 were previously identified in the original Bp genome annotation [15], which also reported an additional 81 other pseudogenes. These 81 genes lack clear homologs in Bt, and were thus not detected by Psi-Phi, which requires a comparative genomic metric for pseudogene identification. A complete list of these genes is provided in the Additional file 1 (supplementary table six). To better understand the molecular events responsible for these pseudogenes, we grouped the Bp and Bt pseudogenes by their functional inactivation categories, ranging from nonsense mutations, frameshifts, IS insertions, to other insertions or deletions, and truncations (Figure 7). In this analysis, "frameshifts" were classified as very small insertions or deletions (typically only 1 or 2 nt in length), "other insertions" were >10 bp in length, and "truncations" included large deletions at either or both the beginning and end of an ORF. Interestingly, when we considered all recognized pseudogenes including those previously annotated and those newly identified, the Bp and Bt genomes were found to display remarkably different mutational spectra. Specifically, internal stop codons due to nonsense mutations were the most usual disruption (~74%) in the Bt genome, followed by truncations (~21%) and frameshifts (~3%). In contrast, a large proportion of the Bp pseudogenes resulted from truncations (~51%), followed by nonsense mutations (~26%) and frameshifts (~17%). In both species, only a few pseudogenes corresponded to IS element insertions.

Around one third of the pseudogenes in both species corresponded to hypothetical proteins and/or proteins of unknown function. However, among the annotated pseudogenes in the Bt genome, there was a distinct non-random functional trend for proteins exhibiting such inactivation events, as they largely involved membrane-associated and exported proteins (26 Bt pseugodgenes, p < 0.001 by Fisher's Test). This bias may reflect the results of strong selective pressures in the natural environments directly contacted by these bacteria. Intriguingly, in the Bp genome, one frequently involved class of proteins that were subject to inactivation events were genes involved in transcription (21 from Bp; p < 0.001), which could lead to differences in activating upstream stimuli and the selection of target genes. This result suggests that certain aspects of Bp-specific behavior may result from alterations in gene expression, in addition to the other large-scale and protein differences previously discussed.

### Comparison of Bp and Bt intergenic regions

Finally, our finding that a number of transcriptional regulators might be targeted for inactivation in Bp made us also investigate the extent to which the transcriptional regulation machinery in Bp and Bt might be similar or different. As a surrogate for gene expression information, we compared intergenic regions of the orthologous protein pairs, which are likely to contain important *cis*-acting promoter elements and motifs for transcriptional regulator docking and activity. Using a working criterion that an intergenic region should be greater than 30 bp and lie between two distinct ORFs, we identified 1191 (~58%) orthologous intergenic regions in Chromosome 1 and 634 (~49%) in Chromosome 2 for further analysis. When assessed at a global level, the intergenic regions of Bp and Bt appear to be similar, in terms of average sequence identity (93% for integenic pairs in both chromosomes), mean lengths, and size ranges (p > 0.05). The high levels of similarity between the intergenic regions could mean that either the transcriptional regulatory machineries of Bp and Bt are highly similar, or that insufficient time has passed for conserved promoter motifs to emerge against the background mutation rate. To test the genomes for any evidence of increased conservation in the intergenic regions, we asked if there was any relationship between the extent of protein similarities between homologs and the degree of sequence conservation in their 5' intergenic regions. Comparing the orthologous gene pairs, we identified a weak but significant positive correlation between protein similarity and adjacent 5' intergenic sequences for genes associated with core metabolism (r = 0.134, P < 0.01, Spearman's correlation coefficient). This bias became stronger particularly for Chromosome 1 when it was treated as separate from Chromosome 2 (r = 0.147, P < 0.0001 for Chromosome 1; r = 0.084, P < 0.05 for Chromosome 2, Spearman's correlation coefficient). In addition, the percentage similarity for promoters of core genes was significantly higher compared to the genome average (p < 0.001, Figure 6b). The finding that orthologs involved in core metabolism already tend to have more conserved 5' intergenic region suggests that some degree of selection in the integenic regions has already taken place. In contrast, for the virulence genes, such a relationship was not observed (p > 0.05). Thus, for genes that are present in both species, the high levels of similarity between their intergenic regions suggest that the *cis*-acting promoter elements of Bp and Bt, are likely to be broadly similar.

## Discussion

Genomic comparisons between pathogens and related nonpathogenic relatives have played an important role in identifying the mechanisms responsible for acquisition of virulence in the natural environment [3,36-39]. Through these analyses, a general picture is now emerging where different pathogens appear to have employed slightly different evolutionary mechanisms to develop virulence. For example, genomic comparisons between pathogenic and laboratory strains of *E. coli *have revealed evidence of a common core chromosome interrupted by the horizontal introduction of multiple segments of virulence related genes (pathogenicity islands) [37,40]. In contrast, the loss of ancestral genomic DNA may play an important role for generating virulence in *Listeria *and *Bordetella *species [38,41]. In the case of *Yersinia pestis*, it has also been proposed that extensive chromosomal rearrangements and massive gene inactivation can also act as a driving force for pathogen evolution [5].

In the case of Bp, our comparative analysis indicates gene mutation, gene deletion, and gene acquisition are likely to represent the major evolutionary drivers of Bp virulence, and that other proposed mechanisms of pathogen evolution, including chromosomal rearragement and bacteriophage-mediated recombination [5,40] may thus a less relevant role in the pathogenic evolution of Bp. Our results are broadly consistent and support the findings of a previous study where genes found to be transcriptionally regulated in *B. mallei *upon infection were compared to their counterparts in Bp and Bt [13]. In that study, the investigators found that the three organisms all possessed the same genome structure of two chromosomes and high levels of conserved nucleotide identity. However, down-regulated genes, which were related to cell growth, were more well conserved while up-regulated potential virulence encoding genes were less well conserved or absent in Bt. Besides confirming these findings on a genome-wide scale, our study also possesses a number of novel features. Specifically, these include I) the discovery and validation of GIs in the Bt genome as genomic elements of lateral transfer, II) that unlike *B. mallei*, the Bp and Bt genomes are highly syntenic, III) the increased divergence of virulence genes, especially those associated with Type III secretion, between Bp and Bt, IV) functional biases in inactivated genes for membrane-associated proteins in Bt and transcription factors in Bp, V) effects of species-specific genes on metabolism and virulence, and VI) evidence that the *cis*-transcriptional regulatory machineries of Bp and Bt are likely to be broadly similar.

To develop as a successful pathogen, virulent bacteria need to evolve both offensive (eg. adherence, invasion, toxin, secretion systems) and defensive pathways (eg. antiphagocytosis, anti-proteolysis, phase variation, serum resistance). Bp and Bt share a large proportion of both offensive and defensive virulence factors (~71%), including adhesion factors, type IV pili, and two Type III secretion systems (TTS2 and TTS3). However, when treated collectively, these virulence genes appear to be significantly more divergent between Bp and Bt compared to the core metabolic genes or the rest of the genome. In contrast to this proteomic comparison, our analysis of the promoters of these genes failed to demonstrate an increased rate of divergence in *cis*-acting loci that might affect the transcriptional regulation of these genes. This result is unlikely to be caused by a lack of sensitivity in our comparison, as we were able to detect a significantly increased rate of conservation in the promoters of genes associated with core metabolism. Thus, at present, we favor the possibility that the *cis*-acting loci responsible for the regulation of these genes are likely to be fairly similar between Bp and Bt. However, we note that a different scenario may pertain to the *trans*-acting loci, since a significant enrichment of transcription factors appear to have been mutationally inactivated or altered in Bp. A close comparison of the transcriptomes of Bp and Bt, which is currently underway in our laboratory, should prove valuable in addressing this issue.

Our results also support key roles for large-scale gene loss, acqusition, and replacement in the development of Bp virulence. For example, both Bp and Bt share the TTS3 Type III secretion system, which is required for the full virulence of Bp in a hamster model of infection [42]. However, it has been recently shown that arabinose exposure may downregulate TTS3 expression and activity [25]. The absence of an arabinose assimilation operon in Bp might thus have contributed to the increased virulence of this species. Besides gene deletion, several gene clusters related to fimbriae and capsular polysaccharides synthesis have also been horizontally transferred to Bp, potentially contributing to the variation of surface components between the two organisms. It has long been recognized that bacterial surface components can play an indispensable role in the pathogenesis of infectious disease, and surface components of Bp may serve as virulence factors by playing a role in the attachment of bacteria to the host cell surface [43,44]. One striking result from our analysis was our discovery that the polysaccharide capsule gene cluster, which has been shown to be an essential virulence determinant [29], is likely to be non-randomly transferred into the Bp genome by replacing a pre-existing gene cluster in Bt already dedicated to polysaccharide synthesis. Polysaccharide coats play an important role in bacterial survival and persistence in the environment and evasion of host immune response, but may not constitute offensive attack. The fact that Bp has higher invasion, adherence capacity, and resistance to phagocytosis are thought to be related to the ability of Bp to produce exopolysaccharides [28,45].

We conclude this report by noting that *B. pseudomallei *is listed as a category B agent on the Centers for Disease Control Bioterrorism Agents/Diseases list [9], and experimental manipulation of Bp is mandated by law in several countries to be conducted under biosafety level 3 (BSL3) laboratory requirements. As many centers in Bp-endemic areas do not have BSL3 facilities, this requirement has somewhat hampered the progress of research in basic Bp biology. By contrast, Bt is considered a risk group 1 agent, and although not considered clinically significant, Bt has been shown to be lethal to the model system *C. elegans *[46]. Thus, although it is undoubtedly essential to be cautious when extrapolating findings from one species to another, the high degree of similarity between the Bt and Bp genomes raises the possibility that Bt could be used as a model system for studying certain aspects of Bp biology, similar to *B. cereus *and *B. anthracis*. The availability of an easily tractable experimental organism, which can be manipulated under standard laboratory conditions, could thus prove useful in accelerating research in the pathogenesis of melioidosis.

## Conclusion

A comparative genomic analysis of Btto Bp has revealed that their molecular inventories are highly similar, in terms of genome structure, gene order, and functional content. Bt contains at least fifteen genomic islands that are variably present across different Bt isolates, whichmay contribute to the presence of different strain-specific phenotypes. Our results suggest that a limited number of horizontal-acquisition events, coupled with the fine-scale functional modulation of existing proteins, are likely to be the major drivers underlying Bp virulence. The extensive genomic similarity between Bp and Bt suggests that, in some cases, Bt could be used as a possible model system for studying certain aspects of Bp behavior.

## Methods

### Phylogenetic analysis

16S ribosomal sequences for *Burkholderia (B. pseudomallei *K96243, *B. thailandensis *E264, *B. mallei *ATCC23343 and *B. cepacia *J2315), and other spp. (*Pseudomonas. aeruginosa *LMG1242T, *Escherichia coli *K12 and *Salmonella typhimurium *LT2) were aligned and compared using MEGA version 3.0 software [47]. Sequence divergence rates were calculated using a neighbor joining algorithm and a bootstrap value of 2000. *P. aeruginosa *LMG1242T was used as an outlier subgroup.

### Bacterial strains and genome sequencing

Two independent cultures of *B. thailandensis *E264, derived from the same original isolate but stored at independent centers (DMERI and USAMRIID), were processed for genomic DNA extraction and whole-genome shotgun (WGS) sequencing at separate facilities (The Institute for Genomic Research (TIGR) and the Broad Institute (BI)). At TIGR, sequencing and assembly followed by genome closure was performed as described in Nierman et al. [17]. Identification of *B. thailandensis *coding sequences (CDs) was performed using GLIMMER (48) with modifications described in Nierman et al. [17]. The BI sequencing approach is highly similar and described in the Main Text and Additional file 4. Our comparative genomic analysis is primarily based on the TIGR E264 genome after incorporating corrections for specific sequencing errors. The TIGR Bt E264 genome sequence has been assigned GenBank accession no. CP000086 and CP0000865. The Whole Genome Shotgun project described in this paper has been deposited at DDBJ/EMBL/GenBank under the project accession AACX00000000. The version described in this paper is the first version, AACX01000000.

### Genome alignments and comparative genomics

The genome sequence of virulent *B. pseudomallei *K96243 was obtained from NCBI [15]. Syntenic regions between the *B. thailandensis *(Bt) and *B. pseudomallei *(Bp) genomes were identified and aligned using MUMMER [49], and visualized using the ARGO Genome Browser [50] (Broad Institute, MIT). Protein homologs between the two species were identified using BLASTP at an E-value threshold of < 10^-10 ^with subsequent confirmation by reciprocal blast. The BLASTP output was also manually curated to generate the final list of orthologous gene pairs between the two species. Pathway comparisons were performed by interrogating the Bp and Bt genomes against the KEGG metabolic database. Fisher's exact test was used to calculate the difference between proportions of functional enrichment. Rates of nucleotide substitution in CDs were calculated by aligning the nucleotide sequences of orthologous gene pairs by Clustalw [51] and determining the number of synonymous nucleotide substitutions per synonymous site (*Ks*) and the number of nonsynonymous nucleotide substitutions per nonsynonymous site (*Ka*) by a maximum-likelihood method [52] in PAML [53]. Spearman's rank correlation analysis was performed using GraphPad Prism version 4.0 (GraphPad Inc., Calif.). Intergenic sequences of orthologous gene pairs, defined as the non-coding sequence between the translational start or stop of two successive genes, that were greater than 30 nucleotides between two species were compared by BLASTN. Two-sample t-tests (for normal distributions) and Mann-Whitney tests (for non-normal distributions) were used to determine the difference between means of different populations.

### Experimental validation of genomic islands

The presence of absence of 15 GIs was assessed across 29 Bt isolates using PCR. The 29 isolates were collected from independent soil locations in two major areas: Northeast Thailand (21 isolates) and Central Thailand (8 isolates). One target gene and one flanking gene were selected for each genomic island based on Bt E264 sequence data. The target genes chosen were predominantly hypothetical conserved proteins; and genes such as integrases and bacteriophage were avoided. The flanking genes were the genes immediately abutting each putative island. The PCR primers and cycling conditions (supplementary table seven in Additional file 1) for the target and flanking genes were optimized using Bt E264 genomic DNA. PCR amplifications were performed with a PTC-0220 DNA engine (MJ Research, Cambridge, MA) with Platinum *Taq *polymerase (Invitrogen), and aliquots of reaction mixtures were analyzed by agarose gel electrophoresis.

### SUPERFAMILY assignment

SUPERFAMILY (release 1.69) is a library of HMM models based on SCOP and contains 1539 known structural superfamilies [33]. The HMM based assignment tool provides structural assignments to protein sequences at the super-family level using known structural information. SUPERFAMILY was run to assign structural superfamilies and domains for Bt and Bp protein sequences in HMMER mode.

### Identifying pseudogenes in Bp and Bt

To identify pseudogenes, we first used protein sequences of Bp (or Bt) genome to query the nucleotide sequence of the Bt (or Bp) genome using TBLASTN. We then applied the Psi-Phi program suite [36] on the BLAST outputs to recover candidate pseudogenes in each genome. Briefly, Psi-Phi is designed to identify pseudogenes by a comparative analysis of related genomes, and retrieves pseudogenes resulting from nonsense mutations, frameshifts generated by small insertions or deletions, large insertions (such as those resulting from transposable elements), and truncations of any specified length as well as any incorrectly annotated spacers resulting from gene degradation [36]. Since Bp and Bt are closely related, a stringent cutoff of E-value < 10^-15 ^and a minimal percentage of protein identity of 79% was used to identity pseudogenes. The candidate pseudogenes were manually curated, and the disrupting mutations determined by aligning the nucleotide sequences of putative pseudogenes with their functional counterparts using CLUSTALW 1.8 [51]. The IS elements were searched by blasting genome sequences against the IS nucleotide database [54].

## Authors' contributions

YY conceived of the study, performed the major analyses, and drafted the manuscript. HHC and SHS performed validation experiments, and CHL and LD contributed towards bioinformatic analyses. Access to Bt E264 genome sequence was provided by HSK, DD, and WCN, and Bt ATCC700388 genome sequence was provided by AD, RE, and BB. PT participated in the design and coordination of the study and manuscript preparation. All authors have read and approved the manuscript.

## Supplementary Material

Additional File 1 Supplementary figures and tables. Click here for file

Additional File 2 Growth curves of *B. thailandensis *E264 and ATCC700388 strains. Click here for file

Additional File 3 Sequence differences between the *B. thailandensis *E264 and ATCC700388 genomes. Click here for file

Additional File 4 Genome sequence of Bt E264 (Broad Institute). Click here for file

## Figures and Tables

**Table 1:** Genome features of Bt and Bp

Feature	Bp		Bt	
	Chromosome 1	Chromosome 2	Chromosome 1	Chromosome 2
Size (bp)	4074542	3173005	3809201	2914771
No. of genes	3460	2394	3282	2363
Percentage coding	3454/3460	2394/2394	3282/3344	2363/2373
Average gene length (nt)	1177	1325	1139	1228
%G + C content	67.7%	68.5%	67.3%	68%
tRNA	53	8	52	6
rRNA	9	3	9	3
No. of Specific genes	484	370	312	339
No. of conserved genes	2970	2024	2970	2024

**Table 2:** Five examples of genomic difference from three categories: Bp-specific; Bt-specific and Bp-Bt divergent. Genes associated with genomic islands are not included in this table

**Bp-specific genes**				**Bt-specific genes**				**Bp-Bt divergent genes**							
**Bp genes**	**Functional notes**	**Size (Kb)**	**GC (%)**	**Bt genes**	**Functional notes**	**Size (Kb)**	**GC (%)**	**Bt genes**	**Functional notes of Bt gene**	**Size (Kb)**	**GC (%)**	**Bp genes**	**Functional notes of Bp gene**	**Size (Kb)**	**GC (%)**

BPSS0995-	Secondary metabolites	69	71.7	BTH_II1662-	Polyketide biosynthesi	89	73	BTH_I2687-	Transposase; unknown	12.6	57.4	BPSL2036-	Unknown function	6.5	55.4
BPSS1011	biosynthesis; lipid transport and metabolism genes			BTH_II1682	s proteins^+^			BTH_I2692	proteins			BPSL2040	genes		
BPSS2210-	Miscellaneous clusters,	60	71.8	BTH_II0143-	Flagella system,	58	65	BTH_II1612-	Miscellaneous clusters,	12.4	66	BPSS0779-	Miscellaneous clusters,	11.2	70.9
BPSS2263	including lipopolysaccharides biosynthesis genes; signal transduction mechanisms genes; etc.			BTH_II0197	chemotaxis-assoicated proteins^+^			BTH_II1622	including Energy production and conversion genes; transcriptional regulators etc.			BPSS0784	including Secondary metabolites biosynthesis; energy production and conversion genes		
BPSS1632	Probable non-ribosomal	53	67.1	BTH_II0913-	Miscellaneous clusters	45.2	69	BTH_II0079-	Transposase	9.8	69.1	BPSS0068-	Transposase; regulator	8.1	65.1
BPSS1640	peptide synthetase (NRPS) cluster			BTH_II0943				BTH_II0083				BPSS0074	protein		
BPSS1375-	Type III systems 1	47	64.3	BTH_II0054-	RND multi-drug efflux	28.4	65	BTH_I1328-	GDP-D-rhamnose	9.7	66.2	BPSL2793-	Capsule biosynthesis	13.9	57.1
BPSS1412	(TTS1)*			BTH_II0072	proteins			BTH_I1335	biosynthesis^+^			BPSL2806	proteins*		
BPSL2848-	Phosphonate utilization	12	70.9	BTH_II1626-	L-arabinose assimilation	10.4	71	BTH_II0431-	Transposase	6.8	62.4	BPSS1940-	Signal transduction;	4.4	69.4
BPSL2860	operon^+^			BTH_II1633	operon *			BTH_II0436				BPSS1941	chemotaxis		

* genes associated with this functional difference have been reported.

^+ ^genes associated with this functional difference are firstly reported

## References

[B1] Walsh C (2000). Molecular mechanisms that confer antibacterial drug resistance. Nature.

[B2] Scarselli M, Giuliani MM, Adu-Bobie J, Pizza M, Rappuoli R (2005). The impact of genomics on vaccine design. Trends Biotechnol.

[B3] Baar C, Eppinger M, Raddatz G, Simon J, Lanz C, Klimmek O, Nandakumar R, Gross R, Rosinus A, Keller H, Jagtap P, Linke B, Meyer F, Lederer H, Schuster SC (2003). Complete genome sequence and analysis of *Wolinella succinogenes*. Proc Natl Acad Sci.

[B4] Dobrindt U, Hochhut B, Hentschel U, Hacker J (2004). Genomic islands in pathogenic and environmental microorganisms. Nat Rev Microbiol.

[B5] Chain PS, Carniel E, Larimer FW, Lamerdin J, Stoutland PO, Regala WM, Georgescu AM, Vergez LM, Land ML, Motin VL, Brubaker RR, Fowler J, Hinnebusch J, Marceau M, Medigue C, Simonet M, Chenal-Francisque V, Souza B, Dacheux D, Elliott JM, Derbise A, Hauser LJ, Garcia E (2004). Insights into the evolution of *Yersinia pestis *through whole-genome comparison with *Yersinia pseudotuberculosis*. Proc Natl Acad Sci USA.

[B6] Vedros NA, Chow D, Liong E (1988). Experimental vaccine against *Pseudomonas pseudomallei *infections in captive cetaceans. Dis Aquat Org.

[B7] Dance DAB (1991). Melioidosis: the tip of the iceberg. Clin Microbiol Rev.

[B8] Yabuuchi E, Arakawa M (1993). *Burkholderia pseudomallei *and melioidosis: be aware in temperate area. Microbiol Immunol.

[B9] Rotz LD, Khan AS, Lillibridge SR, Ostroff SM, Hughes JM (2002). Public health assessment of potential biological terrorism agents. Emerg Infect Dis.

[B10] Wuthiekanun V, Smith MD, Dance DA, Walsh AL, Pitt TL, White NJ (1996). Biochemical characteristics of clinical and environmental isolates of *Burkholderia pseudomallei*. J Med Microbiol.

[B11] Brett PJ, Deshazer D, Woods DE (1997). Characterization of *Burkholderia pseudomallei *and *Burkholderia pseudomallei*-like strains. Epidemiol Infect.

[B12] Brett PJ, DeShazer D, Woods DE (1998). *Burkholderia thailandensis *sp. nov., a *Burkholderia pseudomallei*-like species. Int J Syst Bacteriol.

[B13] Kim HS, Schell MA, Yu Y, Ulrich RL, Sarria SH, Nierman WC, DeShazer D (2005). Bacterial genome adaptation to niches: Divergence of the potential virulence genes in three *Burkholderia *species of different survival strategies. BMC Genomics.

[B14] Cummings CA, Brinig MM, Lepp PW, van de Pas S, Relman DA (2004). *Bordetella *species are distinguished by patterns of substantial gene loss and host adaptation. J Bacteriol.

[B15] Holden MT, Titball RW, Peacock SJ, Cerdeno-Tarraga AM, Atkins T, Crossman LC, Pitt T, Churcher C, Mungall K, Bentley SD, Sebaihia M, Thomson NR, Bason N, Beacham IR, Brooks K, Brown KA, Brown NF, Challis GL, Cherevach I, Chillingworth T, Cronin A, Crossett B, Davis P, DeShazer D, Feltwell T, Fraser A, Hance Z, Hauser H, Holroyd S, Jagels K, Keith KE, Maddison M, Moule S, Price C, Quail MA, Rabbinowitsch E, Rutherford K, Sanders M, Simmonds M, Songsivilai S, Stevens K, Tumapa S, Vesaratchavest M, Whitehead S, Yeats C, Barrell BG, Oyston PC, Parkhill J (2004). Genomic plasticity of the causative agent of melioidosis, *Burkholderia pseudomallei*. Proc Natl Acad Sci USA.

[B16] Coenye T, Vandamme P (2003). Diversity and significance of *Burkholderia *species occupying diverse ecological niches. Environ Microbiol.

[B17] Nierman WC, DeShazer D, Kim HS, Tettelin H, Nelson KE, Feldblyum T, Ulrich RL, Ronning CM, Brinkac LM, Daugherty SC, Davidsen TD, Deboy RT, Dimitrov G, Dodson RJ, Durkin AS, Gwinn ML, Haft DH, Khouri H, Kolonay JF, Madupu R, Mohammoud Y, Nelson WC, Radune D, Romero CM, Sarria S, Selengut J, Shamblin C, Sullivan SA, White O, Yu Y, Zafar N, Zhou L, Fraser CM (2004). Structural flexibility in the *Burkholderia mallei *genome. Proc Natl Acad Sci USA.

[B18] Ochman H, Wilson AC (1987). Evolution in bacteria: evidence for a universal substitution rate in cellular genomes. J Mol Evol.

[B19] Achtman M, Zurth K, Morelli G, Torrea G, Guiyoule A, Carniel E (1999). *Yersinia pestis*, the cause of plague, is a recently emerged clone of *Yersinia pseudotuberculosis*. Proc Natl Acad Sci.

[B20] Ochman H, Jones IB (2000). Evolutionary dynamics of full genome content in *Escherichia coli*. EMBO J.

[B21] DeShazer D, Brett PJ, Woods DE (1998). The type II O-antigenic polysaccharide moiety of *Burkholderia pseudomallei *lipopolysaccharide is required for serum resistance and virulence. Mol Microbiol.

[B22] Soldo B, Lazarevic V, Karamata D (2002). *tagO *is involved in the synthesis of all anionic cell-wall polymers in Bacillus subtilis 168. Microbiology.

[B23] Woods DE, Jeddeloh JA, Fritz DL, DeShazer D (2002). *Burkholderia thailandensis *E125 harbors a temperate bacteriophage specific for *Burkholderia mallei*. J Bacteriol.

[B24] Smith MD, Angus B, Wuthiekanun V, White NJ (1997). Arabinose assimilation defines a non-virulent biotype of *Burkholderia pseudomallei*. Infect Immun.

[B25] Moore RA, Reckseidler-Zenteno S, Kim H, Nierman W, Yu Y, Tuanyok A, Warawa J, DeShazer D, Woods DE (2004). Contribution of gene loss to the pathogenic evolution of *Burkholderia pseudomallei *and *Burkholderia mallei*. Infect Immun.

[B26] Makino K, Kim SK, Shinagawa H, Amemura M, Nakata A (1991). Molecular analysis of the cryptic and functional phn operons for phosphonate use in Escherichia coli K-12. J Bacteriol.

[B27] Wanner BL, Metcalf WW (1992). Molecular genetic studies of a 10.9-kb operon in Escherichia coli for phosphonate uptake and biodegradation. FEMS Microbiol Lett.

[B28] Kespichayawattana W, Intachote P, Utaisincharoen P, Sirisinha S (2004). Virulent *Burkholderia pseudomallei *is more efficient than avirulent *Burkholderia thailandensis *in invasion of and adherence to cultured human epithelial cells. Microb Pathog.

[B29] Reckseidler SL, DeShazer D, Sokol PA, Woods DE (2001). Detection of bacterial virulence genes by subtractive hybridization: identification of capsular polysaccharide of *Burkholderia pseudomallei *as a major virulence determinant. Infect Immun.

[B30] Maki M, Jarvinen N, Rabina J, Roos C, Maaheimo H, Renkonen R, Pirkko, Mattila (2002). Functional expression of Pseudomonas aeruginosa GDP-4-keto-6-deoxy-D-mannose reductase which synthesizes GDP-rhamnose. Eur J Biochem.

[B31] Latifi A, Winson MK, Foglino M, Bycroft BW, Stewart GS, Lazdunski A, Williams P (1995). Multiple homologues of LuxR and LuxI control expression of virulence determinants and secondary metabolites through quorum sensing in *Pseudomonas aeruginosa *PAO1. Mol Microbiol.

[B32] Rainbow L, Hart CA, Winstanley C (2002). Distribution of type III secretion gene clusters in *Burkholderia pseudomallei*, *B. thailandensis *and *B. mallei*. J Med Microbiol.

[B33] Gough J, Karplus K, Hughey R, Chothia C (2001). Assignment of Homology to Genome Sequences using a Library of Hidden Markov Models that Represent all Proteins of Known Structure. J Mol Biol.

[B34] Marlovits TC, Kubori T, Sukhan A, Thomas DR, Galan JE, Unger VM (2004). Structural insights into the assembly of the type III secretion needle complex. Science.

[B35] Balakirev ES, Ayala FJ (2003). Pseudogenes: are they "junk" or functional DNA?. Annu Rev Genet.

[B36] Lerat E, Ochman H (2004). Psi-Phi: exploring the outer limits of bacterial pseudogenes. Genome Res.

[B37] Hayashi T, Makino K, Ohnishi M, Kurokawa K, Ishii K, Yokoyama K, Han CG, Ohtsubo E, Nakayama K, Murata T, Tanaka M, Tobe T, Iida T, Takami H, Honda T, Sasakawa C, Ogasawara N, Yasunaga T, Kuhara S, Shiba T, Hattori M, Shinagawa H (2001). Complete genome sequence of enterohemorrhagic *Escherichia coli *O157:H7 and genomic comparison with a laboratory strain K-12. DNA Res.

[B38] Glaser P, Frangeul L, Buchrieser C, Rusniok C, Amend A, Baquero F, Berche P, Bloecker H, Brandt P, Chakraborty T, Charbit A, Chetouani F, Couve E, de Daruvar A, Dehoux P, Domann E, Dominguez-Bernal G, Duchaud E, Durant L, Dussurget O, Entian KD, Fsihi H, Garcia-del Portillo F, Garrido P, Gautier L, Goebel W, Gomez-Lopez N, Hain T, Hauf J, Jackson D, Jones LM, Kaerst U, Kreft J, Kuhn M, Kunst F, Kurapkat G, Madueno E, Maitournam A, Vicente JM, Ng E, Nedjari H, Nordsiek G, Novella S, de Pablos B, Perez-Diaz JC, Purcell R, Remmel B, Rose M, Schlueter T, Simoes N, Tierrez A, Vazquez-Boland JA, Voss H, Wehland J, Cossart P (2001). Comparative genomics of *Listeria *species. Science.

[B39] Rasko DA, Ravel J, Okstad OA, Helgason E, Cer RZ, Jiang L, Shores KA, Fouts DE, Tourasse NJ, Angiuoli SV, Kolonay J, Nelson WC, Kolsto AB, Fraser CM, Read TD (2004). The genome sequence of *Bacillus cereus *ATCC 10987 reveals metabolic adaptations and a large plasmid related to *Bacillus anthracis *pXO1. Nucleic Acids Res.

[B40] Welch RA, Burland V, Plunkett G, Redford P, Roesch P, Rasko D, Buckles EL, Liou SR, Boutin A, Hackett J, Stroud D, Mayhew GF, Rose DJ, Zhou S, Schwartz DC, Perna NT, Mobley HL, Donnenberg MS, Blattner FR (2002). Extensive mosaic structure revealed by the complete genome sequence of uropathogenic *Escherichia coli*. Proc Natl Acad Sci USA.

[B41] Parkhill J, Sebaihia M, Preston A, Murphy LD, Thomson N, Harris DE, Holden MT, Churcher CM, Bentley SD, Mungall KL, Cerdeno-Tarraga AM, Temple L, James K, Harris B, Quail MA, Achtman M, Atkin R, Baker S, Basham D, Bason N, Cherevach I, Chillingworth T, Collins M, Cronin A, Davis P, Doggett J, Feltwell T, Goble A, Hamlin N, Hauser H, Holroyd S, Jagels K, Leather S, Moule S, Norberczak H, O'Neil S, Ormond D, Price C, Rabbinowitsch E, Rutter S, Sanders M, Saunders D, Seeger K, Sharp S, Simmonds M, Skelton J, Squares R, Squares S, Stevens K, Unwin L, Whitehead S, Barrell BG, Maskell DJ (2003). Comparative analysis of the genome sequences of *Bordetella pertussis, Bordetella parapertussis *and *Bordetella bronchiseptica*. Nat Genet.

[B42] Warawa J, Woods DE (2005). Type III secretion system cluster 3 is required for maximal virulence of *Burkholderia pseudomallei *in a hamster infection model. FEMS Microbiol Lett.

[B43] Ahmed K, Enciso HD, Masaki H, Tao M, Omori A, Traravichikul P, Nagatake T (1999). Attachment of *Burkholderia pseudomallei *to pharyngeal epithelial cells: a highly pathogenic bacteria with low attachment ability. Am J Trop Med Hyg.

[B44] Brown NF, Boddey JA, Flegg CP, Beacham IR (2002). Adherence of *Burkholderia pseudomallei *cells to cultured human epithelial cell lines is regulated by growth temperature. Infect Immun.

[B45] Reckseidler-Zenteno SL, DeVinney R, Woods DE (2005). The capsular polysaccharide of *Burkholderia pseudomallei *contributes to survival in serum by reducing complement factor C3b deposition. Infect Immun.

[B46] O'Quinn AL, Wiegand EM, Jeddeloh JA (2001). *Burkholderia pseudomallei *kills the nematode *Caenorhabditis elegans *using an endotoxin-mediated paralysis. Cell Microbiol.

[B47] Kumar S, Tamura K, Nei M (2004). MEGA3: Integrated Software for Molecular Evolutionary Genetics Analysis and Sequence Alignment. Brief Bioinform.

[B48] Delcher AL, Harmon D, Kasif S, White O, Salzberg SL (1999). Improved microbial gene identification with GLIMMER. Nucleic Acids Res.

[B49] Kurtz S, Phillippy A, Delcher AL, Smoot M, Shumway M, Antonescu C, Salzberg SL (2004). Versatile and open software for comparing large genomes. Genome Biol.

[B50] ARGO Genome Browser. http://www.broad.mit.edu/annotation/argo/.

[B51] Thompson JD, Higgins DG, Gibson TJ (1994). CLUSTAL W: improving the sensitivity of progressive multiple sequence alignment through sequence weighting, position-specific gap penalties and weight matrix choice. Nucleic Acids Res.

[B52] Yang Z, Nielsen R (2000). Estimating synonymous and nonsynonymous substitution rates under realistic evolutionary models. Mol Biol Evol.

[B53] Yang Z (1997). PAML: a program package for phylogenetic analysis by maximum likelihood. Comput Appl Biosci.

[B54] Siguier P, Perochon J, Lestrade L, Mahillon J, Chandler M (2006). ISfinder: the reference centre for bacterial insertion sequences. Nucleic Acids Res.

