# Supplementary material for: Genomic patterns of pathogen evolution revealed by comparison of Burkholderia pseudomallei, the causative agent of melioidosis, to avirulent Burkholderia thailandensis
Source: BMC Microbiol. 2006 May 26;6:46. doi: 10.1186/1471-2180-6-46 (PMC1508146; doi:10.1186/1471-2180-6-46)
Supplement: Additional File 1 — Supplementary figures and tables. [file 1471-2180-6-46-S1.doc]

**Additional file 1: Supplementary figures and tables**

**Figure S1 – Phylogenetic tree depicting the taxonomy and estimated times of divergence between *B. thailandensis* and *B. pseudomallei*.**

The phylogenetic tree is based on nucleotide differences of 16S rRNA sequences and was constructed using neighbor-joining. Numbers on branches indicate the percentage of times the node was supported by 2000 boot-strap replicates. The bottom of legend bar indicates the number of nucleotide differences and the top shows the estimated date of separation (million years ago).

**Figure S2 – Bt genome size compared to other sequenced microbial pathogens**

Among the 217 completely sequenced bacterial genomes up to date, Bt (represented in the red column) is in the top 10% of genome sizes.


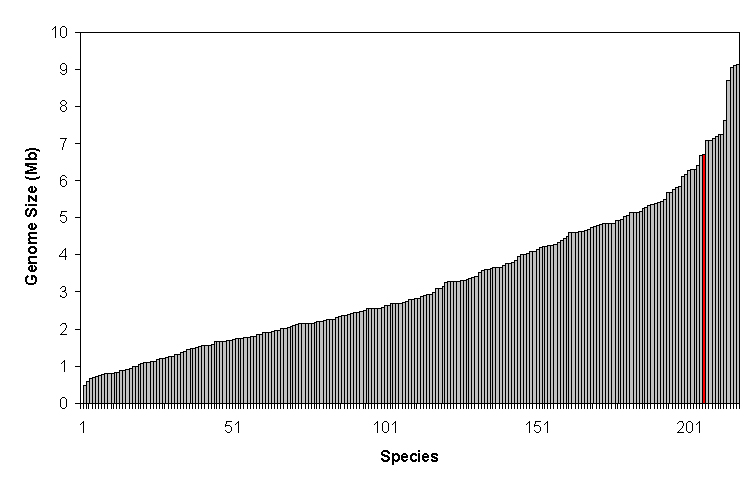


**Table S1. Genomic islands of *B. thailandensis***

| Island | Size, Kb | CDS Coordinates | Integrases | GC (%) | Bp* | Functional note |
| --- | --- | --- | --- | --- | --- | --- |
| **Chromosome 1** |  |  |  |  |  |  |
| GI 1 | 51.3 | BTH_I0091-BTH_I0119 | 1 | + (59.4) | − | Prophage-like |
| GI 2 | 14.4 | BTH_I0909-BTH_I0928 | 1 | − (62.8) | − | Prophage-like |
| GI 3 | 27.1 | BTH_I1439-BTH_I1456 | 0 | + (58.5) | − | Contains transposases and helicase |
| GI 4 | 15.4 | BTH_I1914-BTH_I1932 | 1 | − (62.1) | − | Prophage-like |
| GI 5 | 51.3 | BTH_I2714-BTH_I2748 | 2 | + (58.9) | − | Prophage-like; contains hemolysin activator protein; restriction and modification system |
| GI 6 | 2.7 | BTH_I2889-BTH_I2893 | 0 | + (60.8) | − | Transposases |
| GI 7 | 0.8 | BTH_I2969-BTH_I2971 | 0 | + (59.8) | − | Transposases |
| GI 8 | 21.2 | BTH_I3130-BTH_I3143 | 2 | + (56.5) | + | Contains Transposases, helicases and hypothetical proteins |
| GI 9 | 15.0 | BTH_I3221-BTH_I3231 | − | + (59.7) | + | Miscellaneous island; Contains a Rhs element, transposases and hypothetical proteins |
| GI 10 | 11.3 | BTH_I3266-BTH_I3280 | 1 | + (60.4) | + | Prophage-like |
| **Chromosome 2** |  |  |  |  |  |  |
| GI 11 | 14.7 | BTH_II0349-BTH_II0367 | − | + (59.9) | − | Metabolic island; contains succinate dehydrogenase, carboxymuconolactone decarboxylase and gluconate 5-dehydrogenase |
| GI 12 | 44.7 | BTH_II1011-BTH_II1070 | 1 | − (61.6) | − | Prophage-like |
| GI 13 | 35.5 | BTH_II1325-BTH_II1368 | 0 | − (65.2) | − | Prophage-like |
| GI 14 | 7.3 | BTH_II1526-BTH_II1531 | 1 | − (62.0) | − | Mescellaneous island; Rhs element Vgr protein, |
| GI 15 | 19.3 | BTH_II1996-BTH_II2013 | 2 | + (58.1) | − | Prophage-like |

* Presence or absence of a Bp-GI at this location

**Table S2. Breakpoints of chromosomal rearrangements and flanking genetic elements**

|  | Bt | | Bp | |
| --- | --- | --- | --- | --- |
|  | Coordinates | Flanking genes | Coordinates | Flanking genes |
| Chr 1 | 1245787..2379756 | 3 transposases  3 rRNA | 2506560..3623155 | 3 rRNA |
|  | 3161084..3324521 | 7 transposases  1 recombinase  3 rRNA | 1437846..1614133 | 1 transposase  3 rRNA |
|  | 3701594..3733328 | 2 transposases  2 phage integrases | 3966423..3999388 | 1 phage integrase |
| Chr 2 | 292278..2699557 | 2 transposases | 254427..2874006 |  |

**Table S3 : Non-Syntenic Genomic Regions Between Bp and Bt**

Bp-specific: Present in Bp but absent in Bt

| Bp genes | Functional notes | Size (Kb) | GC (%) |
| --- | --- | --- | --- |
| BPSL0073-BPSL0092 | Bp genomic island 1 | 24.6 | 62.8 |
|
| BPSL0129-BPSL0176 | Bp genomic island 2 | 36.2 | 65.4 |
|
| BPSL0306-BPSL0310 | Function unknown genes | 7 | 70.2 |
|
| BPSL0473-BPSL0494 | Defense mechanism; secondary metabolite biosynthesis genes | 25.2 | 70.9 |
|
| BPSL0548-BPSL0588 | Bp genomic island 3 | 51.1 | 56.6 |
|
| BPSL0680-BPSL0685 | Amino acid transport and metabolism genes | 6.9 | 67.4 |
|
| BPSL0720-BPSL0728 | Energy production and conversion genes | 13.2 | 72.7 |
|
| BPSL0735-BPSL0772 | Bp genomic island 4 | 53.5 | 58.1 |
|
| BPSL0938-BPSL0954 | Bp genomic island 5 | 22.5 | 57.8 |
|
| BPSL1137-BPSL1157 | Bp genomic island 6 | 15.5 | 58.7 |
|
| BPSL1621-BPSL1727 | Bp genomic island 8 | 157.4 | 64.7 |
|
| BPSL2054-BPSL2072 | Signal transduction mechanisms associated genes | 21.7 | 68 |
|
| BPSL2568-BPSL2588 | Function unknown genes | 11.5 | 64.1 |
|
| BPSL2848-BPSL2860 | Phosphonate utilization operon | 12.2 | 70.9 |
|
| BPSL2916-BPSL2920 | Posttranslational modification, protein turnover, chaperones genes | 2.9 | 66.6 |
|
| BPSL3322-BPSL3330 | Lipid transport and metabolism genes; Secondary metabolites transport associated genes | 10.4 | 66.5 |
|
| BPSS0059-BPSS0063 | Signal transduction mechanisms genes | 6.9 | 64.4 |
|
| BPSS0175-BPSS0188 | Function unknown genes | 20.5 | 68.7 |
|
| BPSS0193-BPSS0200 | Amino acid/lipid transport and metabolism genes | 11 | 69.7 |
|
| BPSS0246-BPSS0262 | Ribose transport and metabolism genes | 16.2 | 69.1 |
|
| BPSS0392-BPSS0407 | Bacteriophage protein | 11.7 | 60.7 |
|
| BPSS0433-BPSS0445 | Transcriptional regulators | 16 | 71.1 |
|
| BPSS0605-BPSS0610 | Transcriptional regulators; Secondary metabolites biosynthesis genes | 6.2 | 67 |
|
| BPSS0652A-BPSS0666 | Bp genomic island 14 | 19.2 | 55.3 |
|
| BPSS0734-BPSS0737 | Cell wall/membrane biogenesis genes | 6.8 | 72.1 |
|
| BPSS0762-BPSS0766 | Function unknown genes | 5.8 | 70.8 |
|
| BPSS0796-BPSS0799 | Function unknown genes | 11.9 | 65.7 |
|
| BPSS0819-BPSS0826 | Function unknown genes | 10.8 | 71 |
|
| BPSS0995-BPSS1011 | Secondary metabolites biosynthesis; lipid transport and metabolism genes | 69 | 71.7 |
|
| BPSS1030-BPSS1033 | Carbohydrate transport and metabolism genes | 4.9 | 74.3 |
|
| BPSS1209-BPSS1216 | Function unknown genes | 13.9 | 59.3 |
|
| BPSS1266-BPSS1275 | Secondary metabolites biosynthesis genes | 26.5 | 71.3 |
|
| BPSS1314-BPSS1323 | Function unknown genes | 13.1 | 69.6 |
|
| BPSS1375-BPSS1412 | Type III systems 1 (TTS1) | 46.7 | 64.3 |
|
| BPSS1632-BPSS1640 | Probable non-ribosomal peptide synthetase (NRPS) cluster | 53.1 | 67.1 |
|
| BPSS2046-BPSS2091 | Bp genomic island 16 | 68.2 | 59.6 |
|
| BPSS2117A-BPSS2122 | Function unknown genes | 5.6 | 64 |
|
| BPSS2210-BPSS2263 | Miscellaneous clusters, including lipopolysaccharides biosynthesis genes; signal transduction mechanisms genes; inorganic ion transport and metabolism genes, etc. | 60.4 | 71.8 |
|
| BPSS2289-BPSS2301 | Secondary metabolites biosynthesis genes; Energy production and conversion genes | 19.3 | 70.5 |
|
| BPSS2309-BPSS2312 | Acetyltransferase (GNAT) family protein | 3.9 | 73.1 |
|

Bt-specific: Present in Bt but absent in Bp

| Bt genes | Functional notes | Size  (Kb) | GC (%) |
| --- | --- | --- | --- |
| BTH_I0091-BTH_I0119 | Bt Genomic island 1 | 51.7 | 59.4 |
|
| BTH_I0184-BTH_I0191 | Carbohydrate transport and metabolism genes; Transcriptional regulator; | 9.4 | 69.8 |
|
| BTH_I0909-BTH_I0936 | Bt Genomic island 2 | 22.7 | 62.8 |
|
| BTH_I0998-BTH_I1003 | Transcriptional regulator | 3.3 | 72.5 |
|
| BTH_I1104-BTH_I1101 | Transposase | 2.7 | 61.4 |
|
| BTH_I1364-BTH_I1369 | Function unknown genes | 5.1 | 62 |
|
| BTH_I1439-BTH_I1456 | Bt Genomic island 3 | 28.6 | 58.5 |
|
| BTH_I1914-BTH_I1932 | Bt Genomic island 4 | 17.7 | 62.1 |
|
| BTH_I2687-BTH_I2692 | Function unknown genes | 13.7 | 58 |
|
| BTH_I2714-BTH_I2748 | Bt Genomic island 5 | 52.5 | 58.9 |
|
| BTH_I2889-BTH_I2893 | Transposase | 9.2 | 60.8 |
|
| BTH_I3221-BTH_I3231 | Bt Genomic island 9 | 33.3 | 59.7 |
|
| BTH_I3276-BTH_I3280 | Integrase/transposase | 2.8 | 62.3 |
|
| BTH_II0054-BTH_II0072 | RND multi-drug efflux proteins | 28.4 | 65.2 |
|
| BTH_II0102-BTH_II0110 | Fusaric acid resistance protein; transcriptional regulators | 16.4 | 65.7 |
|
| BTH_II0143-BTH_II0197 | Flagella system, chemotaxis-assoicated proteins | 58 | 65 |
|
| BTH_II0294-BTH_II0236 | Function unknown genes | 15.1 | 64.8 |
|
| BTH_II0316-BTH_II0338 | Energy production and conversion genes; transcriptional regulators | 26.2 | 69.1 |
|
| BTH_II0349-BTH_II0367 | Bt genomic island 11 | 12.5 | 59.4 |
|
| BTH_II0713-BTH_II0721 | Integrase/transposase | 10 | 58.9 |
|
| BTH_II0905-BTH_II0908 | Carbohydrate transport and metabolism genes | 6.6 | 75.3 |
|
| BTH_II0913-BTH_II0943 | Miscellaneous clusters | 45.2 | 69 |
|
| BTH_II1011-BTH_II1070 | Bt genomic island 12 | 44.4 | 61.6 |
|
| BTH_II1075-BTH_II1079 | Drug resistance transporter, EmrB/QacA family; rhamnosyltransferase | 5.1 | 71.5 |
|
| BTH_II1183-BTH_II1190 | Heavy metal efflux pump | 10.9 | 68.8 |
|
| BTH_II1218-BTH_II1222 | Function unknown genes | 5.7 | 71.5 |
|
| BTH_II1291-BTH_II1296 | ABC transporter | 6.3 | 71.5 |
|
| BTH_II1526-BTH_II1531 | Bt genomic island 14 | 7.3 | 62.05 |
|
| BTH_II1526-BTH_II1531 | Transposase; Recombinase | 7.3 | 62 |
|
| BTH_II1626-BTH_II1633 | L-arabinose assimilation operon | 10.4 | 71.1 |
|
| BTH_II1662-BTH_II1682 | Polyketide biosynthesis proteins | 89 | 73.2 |
|
| BTH_II1703-BTH_II1710 | Radical SAM/ThiS domain proteins | 14.1 | 62.2 |
|
| BTH_II1772-BTH_II1779 | cytochrome c; hypothetical proteins | 9.4 | 67.9 |
|
| BTH_II2150-BTH_II2163 | Energy production and conversion genes; transcriptional regulators; Amino acid transport and metabolism genes | 16.3 | 67.4 |
|

Bp-Bt-divergent: Present in Bp and Bt but contents are divergent

| Bt | | | | Bp | | | |
| --- | --- | --- | --- | --- | --- | --- | --- |
| Bt genes | Functional notes of Bt gene | Size  (Kb) | GC | Bp genes | Functional notes of Bp gene | Size  (Kb) | GC |
| BTH_I1328-  BTH_I1335 | Cell wall/membrane biogenesis genes | 9.7 | 66.2 | BPSL2793-  BPSL2806 | Capsule biosynthesis proteins | 13.9 | 57.1 |
| BTH_I2687-  BTH_I2692 | Transposase; unknown proteins | 12.6 | 57.4 | BPSL2036-  BPSL2040 | Unknown function genes | 6.5 | 55.4 |
| BTH_I3128-  BTH_I3143 | Integrase/transposase  Bt genomic island 8 | 24.4 | 56.5 | BPSL3255-  BPSL3270 | Bp genomic island 11 | 18 | 57.1 |
| BTH_I3221-  BTH_I3231 | Bt genomic island 9 | 15 | 59.7 | BPSL0082-  BPSL0092 | Bp genomic island 1 | 10.5 | 61.8 |
| BTH_II0079-  BTH_II0083 | Transposase | 9.8 | 69.1 | BPSS0068-  BPSS0074 | Transposase; regulator protein | 8.1 | 65.1 |
| BTH_II0431-  BTH_II0436 | Transposase | 6.8 | 62.4 | BPSS1940-  BPSS1941 | Signal transduction; chemotaxis | 4.4 | 69.4 |
| BTH_II1612-  BTH_II1622 | Miscellaneous clusters, including inorganic ion transport and metabolism genes; Energy production and conversion genes; transcriptional regulators etc. | 12.4 | 66 | BPSS0779-  BPSS0784 | Miscellaneous clusters, including Secondary metabolites biosynthesis; energy production and conversion genes | 11.2 | 70.9 |

**Table S4. Virulence-associated genes in Bt and their orthologs in Bp**

| Bt Genes | Functional notes | Bp orthologs | Average  Protein Identity |
| --- | --- | --- | --- |
| ***Secretion*** | | | |
| BTH_II0742-BTH_II0759 | Type III protein secretion systems 2 (TTS2) | BPSS1613-BPSS1629 | 74.3% |
| BTH_II0821-BTH_II0830 | TTS3 | BPSS1543-BPSS1552 | 82.6% |
| ***Lipopolysaccarides and capsule*** | | | |
| BTH_I1324-BTH_I1335  BTH_I1338-BTH_I1343 | Capsular polysaccharide synthesis and export cluster | BPSL2787-BPSL2792  BPSL2804-BPSL2810 | 65.8% |
| BTH_I1467-BTH_I1483 | Lipopolysaccarides biosynthetic cluster | BPSL2672-BPSL2688 | 88% |
| BTH_II0543-BTH_II0552 | Potential surface polysaccharide biosynthetic cluster | BPSS1825-BPSS1834 | 85.3% |
| BTH_II1974-BTH_II1986 | Potential surface polysaccharide biosynthetic cluster | BPSS0417-BPSS0429 | 86.8% |
| ***Exoproteins*** | | | |
| BTH_I0317 | Phospholipases C | BPSL0338 | 90% |
| BTH_I1762 | Phospholipases C | BPSL2403 | 95% |
| BTH_II0078 | Phospholipases C | BPSS0067 | 91% |
| BTH_II0379 | Metalloprotease A | BPSS1993 | 74% |
| BTH_I0675 | Ser protease | BPSL0808 | 92% |
| ***Adhesins*** | | | |
| BTH_I0975 | Similar to *Ralstonia solanacearum* putative hemagglutinin-related protein RSP0183 | BPSL1108 | 95% |
| BTH_I2548 | Similar to *R. solanacearum* putative hemagglutinin-related transmembrane protein RSP1094 | BPSL1901 | 45% |
| BTH_II0112 | Hep_Hag family hemagglutinin-like protein | BPSS0088 | 60% |
| BTH_II1489 | Hep_Hag family hemagglutinin-like protein | BPSS0908 | 76% |
| BTH_II0957 | Hep_Hag family hemagglutinin-like protein | BPSS1434 | 78% |
| BTH_II0957 | Similar to C-terminus of *Staphylococcus epidermidis* streptococcal hemagglutinin protein, and to C-terminus of *S. pneumoniae* cell-wall surface anchor family protein | BPSS1439 | 55% |
| BTH_II0398 | C-terminal region similar to *R. solanacearum* putative hemagglutinin-related transmembrane protein, and N-terminal region to *Mycobacterium tuberculosis* hypothetical PE-PGRS family protein precursor. | BPSS1974 | 45% |
| ***Fimbriae and Pili*** | | | |
| BTH_I0649 | Type IV pilin | BPSL0782 | 82% |
| BTH_I0865-BTH_I0866 | Type I fimbriae | BPSL1007-BPSL1008 | 82.5% |
| BTH_I2454-BTH_I2462 | Putative *tad*-type pilus | BPSL1813-BPSL1821 | 76.3% |
| BTH_I2538-BTH_I2546 | Putative *tad*-type pilus | BPSL1891-BPSL1899 | 80% |
| BTH_I2676-BTH_I2681 | Type I fimbriae | BPSL2026-BPSL2031 | 84.8% |
| BTH_I1381-BTH_I1384 | Putative type IV (partial cluster including pilin) | BPSL2752-BPSL2756 | 75.5% |
| BTH_I3025-BTH_I3028 | Putative *tad*-type pilus (partial) | BPSL3170-BPSL3173 | 69% |
| BTH_II0115-BTH_II0118 | Type I fimbriae | BPSS0091-BPSS0094 | 81.7% |
| BTH_II0768-BTH_II0776 | Putative type IVB pilus | BPSS1593-BPSS1602 | 81% |
| BTH_II2263-BTH_II2276 | Putative *tad*-type pilus | BPSS2185-BPSS2198 | 78.9% |
| ***Secondary metabolite biosynthesis genes*** | | | |
| BTH_I2414-BTH_I2419 | Putative hydroxamate siderophore biosynthesis cluster. Linked putative siderophore regulatory and transport genes. Other genes possibly involved in the production of the siderophore | BPSL1774-BPSL1779 | 83% |
| BTH_I1952-BTH_I1971 | Putative nonribosomal peptide synthase (NRPS) cluster. Linked to putative efflux transport genes | BPSL2214-BPSL2233 | 83% |
| BTH_II0204 | Putative polyketide synthase (PKS) | BPSS0130 | 90% |
| BTH_II2088-BTH_II2099 | Putative PKS/NRPS cluster, linked to putative transport regulatory and genes | BPSS0299-BPSS0311 | 84.2% |
| BTH_II1929-BTH_II1935 | Possible PKS/NRPS cluster | BPSS0481-BPSS0487 | 84.3% |
| BTH_II1826-BTH_II1833 | Pyochelin siderophore biosynthesis cluster similar to pyochelin biosynthesis cluster in *Pseudomonas aeruginosa*. Linked putative siderophore regulatory and transport genes | BPSS0581-BPSS0588 | 82.1% |
| BTH_II1233-BTH_II1241 | Putative antibiotic PKS/NRPS. Cluster contains putative efflux protein (BPSS1166). May be linked to the cluster below | BPSS1166-BPSS1174 | 81.1% |
| BTH_II1223-BTH_II1226  BTH_II1209-BTH_II1217 | Putative antibiotic PKS/NRPS. May be linked to the cluster above and a *N*-acyl homoserine lactone dependant quorum sensing regulon (BPSS1176 to PSS1180) | BPSS1181-BPSS1184  BPSS1190-BPSS1199 | 80.1% |
| BTH_II0562-BTH_II0572 | Miscellaneous cluster, possibly involved in the biosynthesis of a nonproteinogenic amino acid | BPSS1805-BPSS1815 | 91.4% |
| BTH_II2344-BTH_II2349 | Possible PKS/NRPS cluster | BPSS2324-BPSS2329 | 88.8% |
| ***Drug resistance*** | | | |
| BTH_I0347 | Ambler class B b -lactamase | BPSL0374 | 90% |
| BTH_I0680-BTH_I0682 | AcrAB-OprM multi-drug efflux pump | BPSL0814-BPSL0816 | 87.3% |
| BTH_I2282 | Ambler class B b -lactamase | BPSL1561 | 87% |
| BTH_I2443-BTH_I2445 | AmrAB multi-drug efflux pump | BPSL1802-BPSL1804 | 80.7% |
| BTH_I1949-BTH_I1951 | RND multi-drug efflux pump | BPSL2234-BPSL2236 | 80% |
| BTH_I1686 | NorM multidrug efflux protein | BPSL2468 | 78% |
| BTH_I1429 | Ambler class B b -lactamase | BPSL2708 | 90% |
| BTH_II2104-BTH_II2106 | RND multi-drug efflux pump | BPSS0292-BPSS0294 | 80% |
| BTH_II1791-BTH_II1792 | RND multidrug efflux pump | BPSS0624-BPSS0625 | 83% |
| BTH_II1450 | PenA; Ambler class A b -lactamase | BPSS0946 | 89% |
| BTH_II0462 | Ambler class B b -lactamase | BPSS1915 | 91% |
| BTH_II0373 | Oxa; Ambler class D b -lactamase | BPSS1997 | 92% |
| ***Intracellular Stress*** | | | |
| BTH_I0744 | putative superoxide dismutase | BPSL0880 | 98% |
| BTH_I0859 | putative membrane-attached superoxide dismutase | BPSL1001 | 97% |
| BTH_I1294 | flavohemoprotein | BPSL2840 | 96% |

Table S5. Top twenty superfamilies assigned to Bp and Bt.

| **Bp** | | **Bt** | |
| --- | --- | --- | --- |
| No. of proteins | SCOP Superfamily | No. of proteins | SCOP Superfamily |
| 282 | P-loop containing nucleoside triphosphate hydrolases | 290 | P-loop containing nucleoside triphosphate hydrolases |
| 207 | Winged helix DNA-binding domain | 218 | Winged helix DNA-binding domain |
| 179 | NAD(P)-binding Rossmann-fold domains | 185 | NAD(P)-binding Rossmann-fold domains |
| 155 | Periplasmic binding protein-like II | 161 | Periplasmic binding protein-like II |
| 109 | MFS general substrate transporter | 125 | Homeodomain-like |
| 92 | alpha/beta-Hydrolases | 104 | MFS general substrate transporter |
| 84 | Homeodomain-like | 88 | S-adenosyl-L-methionine-dependent methyltransferases |
| 79 | S-adenosyl-L-methionine-dependent methyltransferases | 84 | alpha/beta-Hydrolases |
| 74 | PLP-dependent transferases | 71 | CheY-like |
| 70 | CheY-like | 63 | PLP-dependent transferases |
| 58 | FAD/NAD(P)-binding domain | 57 | FAD/NAD(P)-binding domain |
| 55 | Thioredoxin-like | 56 | Thioredoxin-like |
| 50 | ATPase domain of HSP90 chaperone/DNA topoisomerase II/histidine kinase | 51 | ATPase domain of HSP90 chaperone/DNA topoisomerase II/histidine kinase |
| 47 | Acetyl-CoA synthetase-like | 46 | ACP-like |
| 46 | Acyl-CoA N-acyltransferases (Nat) | 44 | Acetyl-CoA synthetase-like |
| 45 | UDP-Glycosyltransferase/glycogen phosphorylase | 44 | Acyl-CoA N-acyltransferases (Nat) |
| 42 | Porins | 43 | UDP-Glycosyltransferase/glycogen phosphorylase |
| 41 | ACP-like | 39 | Homodimeric domain of signal transducing histidine kinase |
| 39 | Nucleotide-diphospho-sugar transferases | 38 | Porins |
| 33 | Thiolase-like | 38 | Thiolase-like |

Table S6a. List of the newly discovered pseudogenes overlapping annotated ORFs in Bp

| **Protein ID in Bt** | **Description** | **Protein ID in Bp** | **Mutation** |
| --- | --- | --- | --- |
| BTH_I0229 | hypothetical protein | BPSL0258 | S |
| BTH_I0258 | putative membrane protein | BPSL0288 | S |
| BTH_I0260 | putative outer membrane porin protein | BPSL0289 | F |
| BTH_I0262 | putative membrane protein | BPSL0291 | S |
| BTH_I0283 | putative cation transporting P-type ATPase | BPSL0302 | S |
| BTH_I0301 | putative methyl-accepting chemotaxis protein | BPSL0323 | S |
| BTH_I0307 | putative 2-nitropropane dioxygenase | BPSL0328 | S |
| BTH_I0333 | putative alkaline phosphatase | BPSL0361 | S |
| BTH_I0367 | putative cytidylyltransferase | BPSL0395 | S |
| BTH_I0392 | LysR family regulatory protein | BPSL0418 | S |
| BTH_I0425 | putative membrane protein | BPSL0452 | S |
| BTH_I0442 | ABC transport system ATP-binding protein | BPSL0469 | S |
| BTH_I0470 | ferredoxin | BPSL0517 | S |
| BTH_I0507 | putative membrane protein | BPSL0590 | S |
| BTH_I0536 | putative polysaccharide biosynthesis/export protein | BPSL0619 | S |
| BTH_I0564 | putative acyl-CoA dehydrogenase oxidoreductase protein | BPSL0648 | S |
| BTH_I0633 | putative two-component regulator histidine sensor kinase | BPSL0732 | S |
| BTH_I0665 | fructose-bisphosphate aldolase | BPSL0798 | S |
| BTH_I0676 | hypothetical protein | BPSL0809 | S |
| BTH_I0688 | putative ABC transporter permease component | BPSL0822 | S |
| BTH_I0706 | aldehyde dehydrogenase family protein | BPSL0843 | S |
| BTH_I0748 | putative membrane protein | BPSL0884 | S |
| BTH_I0755 | putative aldo/keto reductase family oxidoreductase | BPSL0892 | S |
| BTH_I0758 | putative pseudouridine synthase | BPSL0895 | S |
| BTH_I0785 | putative amino-acid transport permease protein | BPSL0920 | S |
| BTH_I0822 | putative permease protein | BPSL0964 | S |
| BTH_I0837 | putativenicotinate-nucleotide--dimethylbenzimidazolephosphoribosyltransferase | BPSL0979 | S |
| BTH_I0846 | ParA family ATPase | BPSL0989 | S |
| BTH_I0905 | hypothetical protein | BPSL1050 | S |
| BTH_I0959 | putative membrane protein | BPSL1092 | S |
| BTH_I1084 | putative phosphotransferase | BPSL1235 | S |
| BTH_I2070 | putative ureidoglycolate hydrolase | BPSL2115 | S |
| BTH_I2053 | hypothetical protein | BPSL2133 | S |
| BTH_I2030 | ribosome recycling factor | BPSL2156 | S |
| BTH_I2001 | family M22 non-peptidase homologue | BPSL2185 | S |
| BTH_I1949 | putative membrane protein | BPSL2236 | S |
| BTH_I1863 | sensor kinase protein | BPSL2302 | S |
| BTH_I1862 | response regulator protein | BPSL2303 | S |
| BTH_I1836 | hypothetical protein | BPSL2326 | S |
| BTH_I1825 | putative hlorohydrolase | BPSL2339 | S |
| BTH_I1805 | hypothetical protein | BPSL2361 | S |
| BTH_I1801 | putative regulatory protein | BPSL2365 | S |
| BTH_I0889 | histidine transport system permease protein | BPSL2394 | S |
| BTH_I1749 | putative membrane protein | BPSL2414 | S |
| BTH_I1734 | putative holo-[acyl-carrier protein] synthase | BPSL2425 | S |
| BTH_I1701 | hypothetical protein | BPSL2458 | S |
| BTH_I1635 | P-protein [bifunctional includes: chorismate mutase and prephenate dehydratase | BPSL2518 | S |
| BTH_I1612 | adenosine deaminase | BPSL2539 | S |
| BTH_I1597 | putative membrane protein | BPSL2554 | S |
| BTH_I1583 | hypothetical protein | BPSL2566 | S |
| BTH_I1571 | cell division topological specificity factor | BPSL2595 | S |
| BTH_I1549 | ABC transporter, substrate binding component | BPSL2615 | S |
| BTH_I1458 | 60 kDa chaperonin | BPSL2697 | S |
| BTH_I1416 | LysR-family regultor | BPSL2720 | S |
| BTH_I1297 | putative carbon-nitrogen hydrolase protein | BPSL2837 | S |
| BTH_I1245 | putative lipoprotein | BPSL2901 | S |
| BTH_I1226 | glutamate/aspartate transport system permease protein | BPSL2923 | S |
| BTH_I1206 | ferric uptake regulator | BPSL2943 | S |
| BTH_I1190 | putative exported ribonuclease | BPSL2958 | S |
| BTH_I1153 | putative ribonucleoside reductase | BPSL2992 | S |
| BTH_II0231 | putative siderophore biosynthesis-related protein | BPSS0162 | S |
| BTH_II1894 | hypothetical protein | BPSS0524 | S |
| BTH_II0707 | formate dehydrogenase-O, major subunit | BPSS1667 | S |
| BTH_I0029 | flagellar protein | BPSL0029 | insertion |
| BTH_I0065 | putative lipoprotein | BPSL0065 | T5' |
| BTH_I0177 | hypothetical protein | BPSL0214 | F |
| BTH_I0282 | putative cheavy metal binding protein | BPSL0301 | T5' |
| BTH_I0351 | putative short chain dehydrogenase | BPSL0379 | T5' |
| BTH_I0571 | thioesterase superfamily protein | BPSL0654 | insertion |
| BTH_I0611 | putative galactonate dehydratase protein | BPSL0699 | T5' |
| BTH_I0902 | putative membrane protein | BPSL1046 | F |
| BTH_I2843 | hypothetical protein | BPSL1291 | F |
| BTH_I2841 | putative membrane protein | BPSL1293 | T5' |
| BTH_I2833 | putative ABC transport system, membrane protein | BPSL1301 | T5' |
| BTH_I2828 | putative LacI-family transcriptional regulator | BPSL1304 | T5' |
| BTH_I2824 | putative membrane protein | BPSL1306 | T5' |
| BTH_I2805 | putative malonate transport-related system membrane protein | BPSL1327 | T5' |
| BTH_I2150 | putative acetyltransferase | BPSL1432 | T5' |
| BTH_I2305 | putative AsnC-family transcriptional regulator | BPSL1588 | F |
| BTH_I2403 | hypothetical protein | BPSL1764 | F |
| BTH_I2492 | hypothetical protein | BPSL1850 | T5' |
| BTH_I2603 | putative LysR-family transcriptional regulator | BPSL1950 | T5' |
| BTH_I2654 | putative thioesterase | BPSL2001 | T5' |
| BTH_I2664 | putative osmosis-related lipoprotein | BPSL2011 | T3' |
| BTH_I2024 | conserved pseudouridylate synthase-related protein | BPSL2162 | T5' |
| BTH_I1818 | hypothetical protein | BPSL2346 | F |
| BTH_I1764 | putative hydrolase | BPSL2401 | T5' |
| BTH_I1674 | dihydrofolate reductase | BPSL2476 | T5' |
| BTH_I1419 | putative lipoprotein | BPSL2717 | T5' |
| BTH_I1401 | putative oxidoreductase | BPSL2735 | T5' |
| BTH_I1274 | putative membrane protein | BPSL2873 | T5' |
| BTH_I1254 | aminopeptidase P | BPSL2892 | T5' |
| BTH_I1249 | crossover junction endodeoxyribonuclease | BPSL2897 | F |
| BTH_I2918 | putative monooxygenase | BPSL3059 | T5' |
| BTH_I2966 | putative lipoprotein | BPSL3109 | T5' |
| BTH_I3100 | phenylacetic acid degradation protein PaaD | BPSL3233 | T5' |
| BTH_I3195 | 30S ribosomal protein S21 | BPSL3318 | T5' |
| BTH_I3255 | aminomethyltransferase | BPSL3360 | T5' |
| BTH_I3341 | hypothetical protein | BPSL3428 | T5' |
| BTH_II0112 | hypothetical protein | BPSS0088 | T5' |
| BTH_II0215 | hypothetical protein | BPSS0146 | T5' |
| BTH_II2194 | hypothetical protein | BPSS0192 | T5' |
| BTH_II2193 | prolyl iminopeptidase | BPSS0201 | T5' |
| BTH_II2184 | hypothetical protein | BPSS0211 | T5' |
| BTH_II2167 | hypothetical protein | BPSS0225 | T5' |
| BTH_II2095 | putative diaminopimelate decarboxylase | BPSS0303 | T5' |
| BTH_II2065 | putative oxidase | BPSS0334 | T5' |
| BTH_II1968 | hypothetical protein | BPSS0446 | T5' |
| BTH_II1757 | putative membrane protein | BPSS0652 | T5' |
| BTH_II1722 | hypothetical protein | BPSS0707 | F |
| BTH_II1714 | hypothetical protein | BPSS0712 | T5' |
| BTH_II1702 | TetR-family regulatory protein | BPSS0718 | T5' |
| BTH_II1540 | hypothetical protein | BPSS0865 | T5' |
| BTH_II1411 | putative membrane protein | BPSS0980 | T5' |
| BTH_II1090 | hypothetical protein | BPSS1334 | T5' |
| BTH_II0971 | hypothetical protein | BPSS1421 | F |
| BTH_II0956 | putative lipoprotein | BPSS1437 | T5' |
| BTH_II0901 | hypothetical protein | BPSS1463 | T5' |
| BTH_II0808 | hypothetical protein | BPSS1565 | T5' |
| BTH_II0763 | hypothetical protein | BPSS1607 | T5' |
| BTH_II0647 | putative membrane protein | BPSS1733 | T5' |
| BTH_II0556 | hypothetical protein | BPSS1821 | T5' |
| BTH_II0469 | putative membrane protein | BPSS1907 | T5' |
| BTH_II0448 | hypothetical protein | BPSS1928 | T5' |
| BTH_II0430 | putative membrane protein | BPSS1942 | T5' |
| BTH_II2281 | hypothetical protein | BPSS2201 | T5' |
| BTH_II2289 | hypothetical protein | BPSS2209 | T5' |
| BTH_II2298 | hypothetical protein | BPSS2268 | T5' |
| BTH_II2321 | HSP20/alpha crystallin family protein | BPSS2288 | T5' |
| BTH_I1004 | hypothetical protein | BPSL1132 | T5' |
| BTH_II2156 | putative squalene/phytoene synthase | BPSS0232 | F |
| BTH_II2081 | putative GntR-family transcriptional regulator | BPSS0320 | insertion |
| BTH_II1853 | putative porin protein | BPSS0562 | S |
| BTH_II1751 | GerR family regulatory protein | BPSS0675 | F |
| BTH_II1644 | hypothetical protein | BPSS0756 | F |
| BTH_II1522 | ArsR family regulatory protein | BPSS0878 | F |
| BTH_II1213 | putative peptide synthase/polyketide synthase | BPSS1194 | insertion |
| BTH_II1213 | putative non-ribosomal peptide synthase | BPSS1195 | insertion |
| BTH_II0963 | hypothetical protein | BPSS1428 | F |
| BTH_II0671 | putative AsnC-family transcriptional regulator | BPSS1710 | F |
| BTH_II0598 | putative MarR-family regulatory protein | BPSS1781 | F |
| BTH_II0516 | hypothetical protein | BPSS1861 | F |
| BTH_II2200 | putative FAA-hydrolase family protein | BPSS2126 | F |
| BTH_II2284 | putative GntR-family regulatory protein | BPSS2204 | F |
| BTH_II2306 | hypothetical protein | BPSS2274 | S |
| BTH_II2361 | squalene/phytoene synthase | BPSS2340 | F |
| BTH_II2368 | hypothetical protein | BPSS2347 | F |

Table 6Sb. List of the new Bp pseudogenes in intergenic regions

| **Protein ID in Bt** | **Description** | **Evalue** | **Strand** | **Position** | | **Mutation** |
| --- | --- | --- | --- | --- | --- | --- |
| BTH_I0311 | Protein of unknown function (DUF1289) family | 2.00E-25 | - | 354839 | 354997 | T5' |
| BTH_I2060 | hypothetical protein | 2.00E-17 | - | 2551292 | 2551507 | T5' |
| BTH_I2623 | hypothetical protein | 2.00E-21 | - | 2345613 | 2345798 | T5' |
| BTH_I2922 | Uncharacterised BCR, putative | 1.00E-46 | - | 3657409 | 3657561 | T3' |
| BTH_I2923 | hypothetical protein | 9.00E-34 | + | 3659226 | 3659411 | T5' |
| BTH_I3289 | hypothetical protein | 1.00E-37 | - | 4008185 | 4008433 | T5' |
| BTH_II1039 | hypothetical protein | 6.00E-21 | - | 539805 | 539963 | T |
| BTH_II1125 | hypothetical protein | 3.00E-17 | - | 1773177 | 1773296 | T3' |
| BTH_II0221 | hypothetical protein | 2.00E-25 | + | 198924 | 199037 | T |
| BTH_I3271 | hypothetical protein | 9.00E-17 | + | 3721761 | 3721892 | T |
| BTH_II1364 | DNA methyltransferase | 2.00E-21 | + | 1429265 | 1429402 | T |
| BTH_I2117 | glycerate kinase 1 | 6.00E-156 | - | 1630024 | 1630199 | T |
| BTH_II2087 | autoinducer-binding transcriptional regulator, | 2.00E-21 | + | 438052 | 438228 | T |
| BTH_II1781 | outer membrane protein, OMP85 family, putative | 4.00E-19 | - | 864101 | 864290 | T |
| BTH_II2147 | L-allo-threonine aldolase | 4.00E-24 | + | 320815 | 321015 | T |
| BTH_II0676 | probable transmembrane protein | 1.00E-30 | - | 2338434 | 2338775 | T |
| BTH_I2075 | Protein of unknown function (DUF1458) family | 2.00E-43 | + | 2535440 | 2535502 | T |
| BTH_I2695 | hypothetical protein | 4.00E-34 | - | 2441979 | 2442071 | T |
| BTH_II1311 | Mg2+-importing ATPase, putative | 4.00E-18 | + | 1475608 | 1475706 | T |
| BTH_I2146 | hypothetical protein | 6.00E-77 | + | 1665603 | 1665701 | T |
| BTH_II0623 | Bbp50 | 2.00E-77 | + | 2406099 | 2406656 | F |
| BTH_II1070 | MFS transporter, putative | 6.00E-47 | + | 1856935 | 1857321 | S |
| BTH_II2007 | splicing coactivator subunit-like protein | 8.00E-36 | - | 525850 | 526053 | S |

Table S6c. List of the pseudogenes overlapping annotated ORFs in Bt

| **Protein ID in Bp** | **Description** | **Protein ID in Bt** | **Mutation** |
| --- | --- | --- | --- |
| BPSL1050 | hypothetical protein | BTH_I0905 | S |
| BPSL1103 | endonuclease III | BTH_I0970 | S |
| BPSL1166 | uracil phosphoribosyltransferase | BTH_I1016 | S |
| BPSL1169 | quinone oxidoreductase | BTH_I1019 | S |
| BPSL1195 | RNA polymerase sigma-70 factor, ECF subfamily | BTH_I1044 | S |
| BPSL1230 | MoaC domain protein | BTH_I1080 | S |
| BPSL1264 | bcpB, putative | BTH_I2870 | S |
| BPSL1276 | iron compound ABC transporter, ATP-binding protein, putative | BTH_I2858 | S |
| BPSL1289 | lipoprotein, putative | BTH_I2845 | S |
| BPSL1300 | ABC transporter, ATP-binding protein | BTH_I2834 | S |
| BPSL1344 | hydroxyacylglutathione hydrolase | BTH_I2788 | S |
| BPSL1356 | cell division protein FtsH | BTH_I2776 | S |
| BPSL1369 | PAP2 family protein | BTH_I2761 | S |
| BPSL1420 | intracellular septation protein A | BTH_I2138 | S |
| BPSL1426 | thiolase family protein | BTH_I2144 | S |
| BPSL1437 | DNA polymerase III, delta prime subunit | BTH_I2155 | S |
| BPSL1454 | cytochrome c oxidase family protein | BTH_I2175 | S |
| BPSL1455 | Ser/Thr protein phosphatase family protein | BTH_I2176 | S |
| BPSL1458 | ribosomal protein S6 | BTH_I2179 | S |
| BPSL1459 | primosomal replication protein n | BTH_I2180 | S |
| BPSL1510 | nucleoside diphosphate kinase | BTH_I2231 | S |
| BPSL1602 | hypothetical protein | BTH_I2320 | S |
| BPSL1603 | protein disulfide isomerase NosL, putative | BTH_I2321 | S |
| BPSL1730 | hypothetical protein | BTH_I2372 | S |
| BPSL1739 | ABC transporter, ATP-binding protein | BTH_I2380 | S |
| BPSL1744 | ornithine carbamoyltransferase | BTH_I2385 | S |
| BPSL1814 | pilus assembly protein, putative | BTH_I2455 | S |
| BPSL1841 | hypothetical protein | BTH_I2482 | S |
| BPSL1865 | arginine-tRNA-protein transferase, putative | BTH_I2510 | S |
| BPSL1873 | RNA methyltransferase, TrmH family, group 3 | BTH_I2518 | S |
| BPSL1883 | long-chain-fatty-acid--CoA ligase | BTH_I2529 | S |
| BPSL1897 | hypothetical protein | BTH_I2544 | S |
| BPSL1898 | peptidase, putative | BTH_I2545 | S |
| BPSL1926 | hypothetical protein | BTH_I2572 | S |
| BPSL1942 | ribosomal protein L20 | BTH_I2592 | S |
| BPSL2029 | Spore Coat Protein U domain family | BTH_I2679 | S |
| BPSL2030 | hypothetical protein | BTH_I2680 | S |
| BPSL2051 | PAAR motif family | BTH_I2706 | S |
| BPSL2086 | hypothetical protein | BTH_I1927 | S |
| BPSL2135 | TGS domain protein | BTH_I2051 | S |
| BPSL2150 | outer membrane protein, OmpH/HlpA family | BTH_I2036 | S |
| BPSL2163 | polypeptide deformylase | BTH_I2023 | S |
| BPSL2196 | acetyltransferase, GNAT family | BTH_I1990 | S |
| BPSL2199 | NLP/P60 family protein | BTH_I1982 | S |
| BPSL2218 | glycosyl transferase, group 2 family protein | BTH_I1967 | S |
| BPSL2268 | hypothetical protein | BTH_I1896 | S |
| BPSL2313 | nitrate/nitrite sensory protein NarX, putative | BTH_I1850 | S |
| BPSL2326 | Protein of unknown function (DUF495) family | BTH_I1836 | S |
| BPSL2340 | imidazolonepropionase | BTH_I1824 | S |
| BPSL2343 | histidine utilization repressor | BTH_I1821 | S |
| BPSL2380 | Cytochrome c oxidase subunit III | BTH_I1786 | S |
| BPSL2424 | glycosyl hydrolase, family 3 | BTH_I1735 | S |
| BPSL2499 | electron transfer flavoprotein, alpha subunit | BTH_I1654 | S |
| BPSL2502 | D-methionine ABC transporter, permease protein | BTH_I1651 | S |
| BPSL2542 | Uncharacterized protein family UPF0016 family | BTH_I1609 | S |
| BPSL2668 | glycosyl transferase, group 2 family protein | BTH_I1487 | S |
| BPSL2673 | glycosyl transferase, group 4 family protein | BTH_I1482 | S |
| BPSL2712 | glycerophosphoryl diester phosphodiesterase family protein | BTH_I1425 | S |
| BPSL2782 | transcriptional regulator, AraC family, putative | BTH_I1350 | S |
| BPSL2878 | hypothetical protein | BTH_I1269 | S |
| BPSL2910 | ribosomal protein S9 | BTH_I1234 | S |
| BPSL2928 | adenylosuccinate lyase | BTH_I1221 | S |
| BPSL2929 | thermoresistant gluconokinase | BTH_I1220 | S |
| BPSS0001 | hypothetical protein | BTH_II0001 | S |
| BPSS0021 | siderophore-interacting protein | BTH_II0023 | S |
| BPSS0031 | transcriptional regulator, Crp/Fnr family | BTH_II0035 | S |
| BPSS0111 | putative cytoplasmic protein | BTH_II0135 | S |
| BPSS0113 | Protein of unknown function (DUF1316) subfamily, putative | BTH_II0137 | S |
| BPSS0289 | hypothetical protein | BTH_II2111 | T5' |
| BPSS0308 | adenosylmethionine-8-amino-7-oxononanoate aminotransferase, putative | BTH_II2091 | S |
| BPSS0346 | ABC transporter, permease protein | BTH_II2052 | S |
| BPSS0465 | putrescine ABC transporter, permease protein | BTH_II1950 | S |
| BPSS0474 | agmatinase, putative | BTH_II1941 | S |
| BPSS0487 | hypothetical protein | BTH_II1929 | S |
| BPSS0497 | Bacterial domain of unknown function (DUF403) superfamily | BTH_II1921 | S |
| BPSS0498 | Transglutaminase-like superfamily domain protein | BTH_II1920 | S |
| BPSS0627 | Ribonuclease T2 family | BTH_II1789 | S |
| BPSS0644 | SIS domain protein | BTH_II1764 | S |
| BPSS0684 | acyl-CoA dehydrogenase domain protein | BTH_II1742 | S |
| BPSS0696 | 5-carboxymethyl-2-hydroxymuconate delta isomerase | BTH_II1731 | S |
| BPSS0704 | ABC transporter, periplasmic substrate-binding protein | BTH_II1725 | S |
| BPSS0708 | outer membrane porin OpcP | BTH_II1720 | S |
| BPSS0837 | universal stress protein family domain protein | BTH_II1568 | S |
| BPSS0882 | CBS domain protein | BTH_II1515 | S |
| BPSS0885 | N-acyl homoserine lactone synthase | BTH_II1512 | S |
| BPSS0907 | major facilitator family transporter | BTH_II1490 | S |
| BPSS0911 | amidase family protein | BTH_II1486 | S |
| BPSS1099 | hypothetical protein | BTH_II1310 | S |
| BPSS1134 | transcriptional regulator, PadR family domain protein | BTH_II1272 | S |
| BPSS1145 | hypothetical protein | BTH_II1263 | S |
| BPSS1153 | hypothetical protein | BTH_II1255 | S |
| BPSS1206 | 2OG-Fe(II) oxygenase superfamily:Prolyl 4-hydroxylase, alpha subunit | BTH_II1201 | S |
| BPSS1324 | 1-aminocyclopropane-1-carboxylate deaminase | BTH_II1101 | S |
| BPSS1338 | YeeE/YedE family protein | BTH_II1086 | S |
| BPSS1461 | DNA-binding response regulator | BTH_II0903 | S |
| BPSS1505 | pentapeptide repeat family protein, putative | BTH_II0861 | S |
| BPSS1550 | type III secretion system BasJ | BTH_II0823 | S |
| BPSS1611 | hypothetical protein | BTH_II0761 | S |
| BPSS1624 | lipoprotein transmembrane protein | BTH_II0747 | S |
| BPSS1667 | formate dehydrogenase, alpha subunit, selenocysteine-containing | BTH_II0707 | S |
| BPSS1713 | glyoxalase family protein family | BTH_II0667 | S |
| BPSS1723 | lyase, putative | BTH_II0657 | S |
| BPSS1748 | hypothetical protein | BTH_II0632 | S |
| BPSS1751 | transcriptional regulator, Sir2 family | BTH_II0629 | S |
| BPSS1752 | cytosine/purines/uracil/thiamine/allantoin permease family protein | BTH_II0628 | S |
| BPSS1765 | iron-sulfur cluster-binding protein, rieske family | BTH_II0611 | S |
| BPSS1766 | rhodanese domain protein | BTH_II0610 | S |
| BPSS1782 | organic hydroperoxide resistance protein | BTH_II0597 | S |
| BPSS1787 | molybdate ABC transporter, permease protein | BTH_II0592 | S |
| BPSS1819 | serine/threonine protein phosphatase 1 | BTH_II0558 | S |
| BPSS1823 | FK506-binding protein | BTH_II0554 | S |
| BPSS1843 | DGPF domain protein | BTH_II0534 | S |
| BPSS1844 | DGPF domain superfamily | BTH_II0533 | S |
| BPSS1879 | acetolactate synthase, putative | BTH_II0497 | S |
| BPSS1890 | transcriptional regulator CatR | BTH_II0486 | S |
| BPSS1893 | muconolactone delta-isomerase | BTH_II0483 | S |
| BPSS1894 | ubiquinol oxidase, subunit IV | BTH_II0482 | S |
| BPSS1897 | ubiquinol oxidase, subunit II | BTH_II0479 | S |
| BPSS1909 | isochorismatase family protein | BTH_II0467 | S |
| BPSS1931 | ABC transporter, ATP-binding protein | BTH_II0445 | S |
| BPSS1952 | ATP synthase F1, epsilon subunit | BTH_II0420 | S |
| BPSS1958 | osmotically inducible protein Y domain protein | BTH_II0414 | S |
| BPSS1994 | DNA-binding response regulator IrlR | BTH_II0376 | S |
| BPSS2019 | conserved within P. aerophilum | BTH_II0313 | S |
| BPSS2098 | Protein of unknown function (DUF796) superfamily | BTH_II0260 | S |
| BPSS2105 | ompA family protein | BTH_II0253 | S |
| BPSS2129 | hypothetical protein | BTH_II2205 | S |
| BPSS2205 | MlrC C-terminus family | BTH_II2285 | S |
| BPSS2206 | major facilitator family transporter | BTH_II2286 | S |
| BPSL0242 | threonine efflux protein, putative | BTH_I0212 | T5' |
| BPSL0890 | citrate synthase family protein | BTH_I0753 | T3' |
| BPSL1368 | hypothetical protein | BTH_I2763 | T5' |
| BPSL1590 | hypothetical protein | BTH_I2307 | F |
| BPSL1799 | fimbrial chaperone protein | BTH_I2442 | T5' |
| BPSS0223 | hypothetical protein | BTH_II2169 | T5' |
| BPSS0270 | transcriptional regulator, LysR family | BTH_II2130 | T5' |
| BPSS0272 | major facilitator family transporter | BTH_II2129 | T5' |
| BPSS0313 | D-methionine ABC transporter, periplasmic D-methionine-binding protein | BTH_II2086 | T5' |
| BPSS0505 | transcriptional regulator, AraC family | BTH_II1912 | T5' |
| BPSS0753 | hypothetical protein | BTH_II1648 | T5' |
| BPSS0967 | hypothetical protein | BTH_II1424 | T5' |
| BPSS0988 | transporter, putative | BTH_II1403 | T3' |
| BPSS1212 | hypothetical protein | BTH_II0094 | T5' |
| BPSS1591 | hypothetical protein | BTH_II0778 | T5' |
| BPSS1631 | syringomycin biosynthesis enzyme, putative | BTH_II0737 | T5' |
| BPSS1676 | hypothetical protein | BTH_II0705 | T3' |
| BPSS2001 | hypothetical protein | BTH_II0368 | T5' |
| BPSS2125 | L-lactate dehydrogenase | BTH_II0240 | T5' |
| BPSS2178 | glyoxalase family protein superfamily | BTH_II2257 | T5' |
| BPSL0362 | hypothetical protein | BTH_I0334 | F |
| BPSL0362 | hypothetical protein | BTH_I0335 | F |
| BPSL1022 | endo/excinuclease domain protein | BTH_I0879 | S |
| BPSL1427 | hypothetical protein | BTH_I2145 | T5' |
| BPSS0064 | hypothetical protein | BTH_II0076 | T5' |
| BPSS0278 | hypothetical protein | BTH_II2123 | S |
| BPSS0961 | hypothetical protein | BTH_II1431 | insertion |
| BPSS0961 | hypothetical protein | BTH_II1432 | insertion |
| BPSS1469a | addiction module antitoxin, Axe family subfamily, putative | BTH_II0893 | T3' |
| BPSS1729 | cytochrome c family protein | BTH_II0652 | insertion |
| BPSS1729 | probable cytochrome c PA4571 | BTH_II0653 | insertion |
| BPSS1900 | transcriptional regulator lysR family | BTH_II0476 | T5' |
| BPSS1998 | hypothetical protein | BTH_II0370 | F |
| BPSS1998 | hypothetical protein | BTH_II0371 | F |

Table 6Sd. List of the new Bt pseudogenes in intergenic regions

| **Protein ID in Bp** | **Description** | **Evalue** | **Strand** | **Position** | | **Mutation** |
| --- | --- | --- | --- | --- | --- | --- |
| BPSL1372 | putative membrane protein | 2.00E-25 | - | 1037767 | 1037937 | T3' |
| BPSL2036 | hypothetical protein | 4.00E-33 | - | 3067760 | 3067966 | T5' |
| BPSL2037 | hypothetical protein | 8.00E-18 | + | 3076401 | 3076481 | T5' |
| BPSL2487 | hypothetical protein | 1.00E-26 | - | 1874055 | 1874258 | T |
| BPSS0230 | Glyoxalase/Bleomycin resistance | 1.00E-24 | + | 2648133 | 2648338 | T3' |
| BPSS1073A | hypothetical protein | 5.00E-35 | - | 1594382 | 1594588 | T |
| BPSL2474B | putative membrane protein | 9.00E-44 | - | 1886141 | 1886470 | T |
| BPSS2166 | hypothetical protein | 9.00E-60 | + | 2753466 | 2753831 | T5' |
| BPSL2125 | hypothetical protein | 5.00E-30 | + | 2334325 | 2334372 | T |
| BPSS0758 | hypothetical protein | 2.00E-52 | - | 1927446 | 1927517 | T |
| BPSS1439 | putative membrane-anchored cell surface protein | 2.00E-42 | - | 1132718 | 1132810 | T |
| BPSL0095 | hypothetical protein | 7.00E-46 | + | 86140 | 86432 | F |
| BPSL0113 | hypothetical protein | 4.00E-26 | - | 97814 | 97987 | T5' |
| BPSL1376 | putative membrane protein | 2.00E-29 | + | 3168780 | 3169031 | S |
| BPSL1583 | hypothetical protein | 4.00E-63 | - | 2595957 | 2596337 | T5' |
| BPSL1900 | hypothetical protein | 6.00E-20 | - | 2903872 | 2904006 | S |

Note: S: stop codon; F: frameshift; T: fragment; T5’: truncated in 5’ end; T3’: truncated in 3’ end; IS: disrupted by IS element; insertion: insertion of nucleotides or genes.

Table S7. Primers and cycling conditions for PCR

| Island target sequence | Gene target | Primer-Forward | Primer-Reverse | Product size |
| --- | --- | --- | --- | --- |
| GI-1 |  |  |  |  |
| Island | BTH_I0097 | AGCCGGTATGGTCTATGTCG | GCGATCCTCAACTGGCTAAG | 237 bp |
| Flanking gene | BTH_I0090 | ATCAATCGGATTCAGGACGA | AGCTTGTCCAACTGCTTGCT | 230 bp |
| GI-2 |  |  |  |  |
| Island | BTH_I0919 | GCGTACCGATCACAGAACCT | TGCAACAGACCGACTTGAAC | 208 bp |
| Flanking gene | BTH_I0937 | GACAAATCCGGTACCATTGC | TATAGACCCAGCCGCAAATC | 152 bp |
| GI-3 |  |  |  |  |
| Island | BTH_I1455 | TGAGCTTGCCATACACTTGC | CTTGGCTACGAAGGATCCAG | 247 bp |
| Flanking gene | BTH_I1438 | GTTGAACACGCTTCCGATTT | CCAGCCAAGTTGTCGAGTG | 168 bp |
| GI-4 |  |  |  |  |
| Island | BTH_I1924 | CGAGCTTCGAGGAAATTCTG | CCTTGGTCTTCGTTCTCGTC | 241 bp |
| Flanking gene | BTH_I1913 | ACAACAAGGTTCACGGCTTC | ACCCTCGATGTGAGGCAATA | 224 bp |
| GI-5 |  |  |  |  |
| Island | BTH_I2720 | CACCTGTTGAGGTCACGATG | GCCTTCTTGCCATCTTTGAG | 214 bp |
| Flanking gene | BTH_I2708 | CCATCTGCTGTTGAAGGACA | GCGTCGACCGAATAGACTTC | 222 bp |
| GI-6 |  |  |  |  |
| Island | BTH_I2889 | TGTCAACAGAAGCACCGAAG | CGACGCCATTTGAATAACAA | 163 bp |
| Flanking gene | BTH_I2899 | ACGAGAACATCCTGGTGGAG | ACGAATAGGTCGCCATCATC | 216 bp |
| GI-7 |  |  |  |  |
| Island | BTH_I2969 | CGTTCGACATACGCATTCTG | TCAACGTGATCGACGACTTC | 244 bp |
| Flanking gene | BTH_I2968 | TCTTCATGCTGTTCCTGCTG | GATCTTGAACTGCGACGACA | 197 bp |
| GI-8 |  |  |  |  |
| Island | BTH_I3133 | AACGGACTCACCGACGATAC | TGCCAACAGTTCACGAAGAC | 242 bp |
| Flanking gene | BTH_I3129 | AAGGTGTTCGTCTGGGTGAC | TTCGCGTATCCGGAAAATAG | 217 bp |
| GI-9 |  |  |  |  |
| Island | BTH_I3227 | CAAGAAGATGCGAACACGAA | TAGGGGCGAATCAATCTGTC | 245 bp |
| Flanking gene | BTH_I3220 | GAAGAGCAGGGGTATGACCA | TCCAGGATGCACGTGTGATA | 155 bp |
| GI-10 |  |  |  |  |
| Island | BTH_I3267 | CGAATGATGTGGACGTGAAG | GCCCCATAACGAAGATGAGA | 219 bp |
| Flanking gene | BTH_I3261 | ACTCATCGCAGTTGTCATCG | GCAGACGGCCTTGTAGACTT | 220 bp |
| GI-11 |  |  |  |  |
| Island | BTH_II0353 | CACGGTCAAGGAAAGACCAT | GCAGCACTTCAAAACGATCA | 151 bp |
| Flanking gene | BTH_II0368 | ACCGCGCAACTGAACATC | TTATTCCCCGATCCGTTCTT | 168 bp |
| GI-12 |  |  |  |  |
| Island | BTH_II1028 | ACGTGGAAGCGTTGGACTAC | CGCGTAGCTTCCGTTCTAAG | 241 bp |
| Flanking gene | BTH_II1010 | TCTACACCGTGCTGATCTCG | CGGCGTATACGTGTAGAGCA | 239 bp |
| GI-13 |  |  |  |  |
| Island | BTH_II1360 | GATGACATGGCGAAATTGCT | TCGATTTCTGCACACGCTAC | 248 bp |
| Flanking gene | BTH_II1324 | GGTGAAGCAGTTCGACATCA | GGCCGAATCAGATAATCGTC | 180 bp |
| GI-14 |  |  |  |  |
| Island | BTH_II1527 | CCGCATCGTCACCTTAATTT | AGCTGCTTTGCTTGCTCTTC | 206 bp |
| Flanking gene | BTH_II1525 | GCGAGTGGCCGCTACTATAC | CGCACACGTAAGCCAGACTA | 239 bp |
| GI-15 |  |  |  |  |
| Island | BTH_II2008 | GCAAGATCACCATCAACACG | GATGCGTTTACGAACGAGGT | 248 bp |
| Flanking gene | BTH_II1995 | GGCGTACACGGTAGGCTATG | CGTGTGGTACCGAAACGTAA | 154 bp |

Notes: Cycling conditions: 30 sec, 94 ºC; 30 sec, 55 ºC; 30 sec, 72 ºC
