# Supplementary material for: Genomic patterns of pathogen evolution revealed by comparison of Burkholderia pseudomallei, the causative agent of melioidosis, to avirulent Burkholderia thailandensis
Source: BMC Microbiol. 2006 May 26;6:46. doi: 10.1186/1471-2180-6-46 (PMC1508146; doi:10.1186/1471-2180-6-46)
Supplement: Additional File 2 — Growth curves of B. thailandensis E264 and ATCC700388 strains. [file 1471-2180-6-46-S2.doc]

**Additional file 2: Growth curves of *B. thailandensis* E264 and ATCC700388 strains**

**1. Cell cultures**

We generated growth curves for *B. thailandensis,* E264 (sequenced at TIGR) and ATCC700388 (sequenced at the Broad Institute) in minimal media (CDM; 10X stock: 0.03M NaCl, 0.032M Mg_2_SO_4_.7H_2_O, 0.03M KCl, 0.03M (NH_4_)_2_SO_4_, 0.012M K_2_HPO_4_, 0.55M Glucose, 0.5M MOPS). A single bacterial colony was inoculated into 5ml TSB (Oxoid, England) and the starter cultures were incubated for 15hrs at 37^0^C with shaking at 70rpm. The cultures were then centrifuged at 3000rpm for 10mins after which the supernatant were discarded and the pellet was washed three times with 1X PBS. The washed bacterial pellet was then diluted with 1X CDM to a final volume of 50ml with OD_600nm_ of 0.05 in 250ml Erlenmeyer flasks. All cultures were then incubated at 37^0^C with shaking at 150rpm. At hourly intervals for up to 24h in 1X CDM, 1ml of culture was taken from each culture and optical density readings (OD_600nm_) were measured spectrometrically with a Ultraspec 10 Cell Density Meter (Amersham Biosciences, Singapore) .

**2. Comparison of growth curves of Bt strains**

This experiment showed that the two strains display different growth rates in CDM. As shown in the figure below, growth of Bt ATCC700388 (pink) lagged behind Bt E264 (blue) during the initial part of log phase. After entering a stationary phase, Bt ATCC700388 (pink) consistently attained higher densities than Bt E264 (blue).

Average growth curves of Bt E264 and ATCC700388 cultures in media CDM (Based on Three Independent Growth Experiments per Strain)
