# Supplementary material for: Genomic patterns of pathogen evolution revealed by comparison of Burkholderia pseudomallei, the causative agent of melioidosis, to avirulent Burkholderia thailandensis
Source: BMC Microbiol. 2006 May 26;6:46. doi: 10.1186/1471-2180-6-46 (PMC1508146; doi:10.1186/1471-2180-6-46)
Supplement: Additional File 3 — Sequence differences between the B. thailandensis E264 and ATCC700388 genomes. [file 1471-2180-6-46-S3.doc]

**Additional file 3: Sequence differences between the *B. thailandensis* E264 and ATCC700388 genomes**

We compared the genome sequences of two distinct isolates of Bt strain E264 sequenced at The Institute for Genomic Research (TIGR), and Bt strain ATCC700388 sequenced at the Broad Institute (BI)). These two genome sequences are referred to as the TIGR and BI sequences. As the BI sequence was not assembled to closure, there are 44 gaps in the BI sequence (29 in Chr 1, 15 in Chr 2). Our comparison is confined to regions that could be confidently matched between the two genomes, thus regions in these sequence gaps were deliberately ignored.

**1. Large-scale differences between the genomes.**

Using methods described in the Main Text, we aligned the TIGR and BI sequences and visualized the alignment in the form of a dot-matrix diagram.


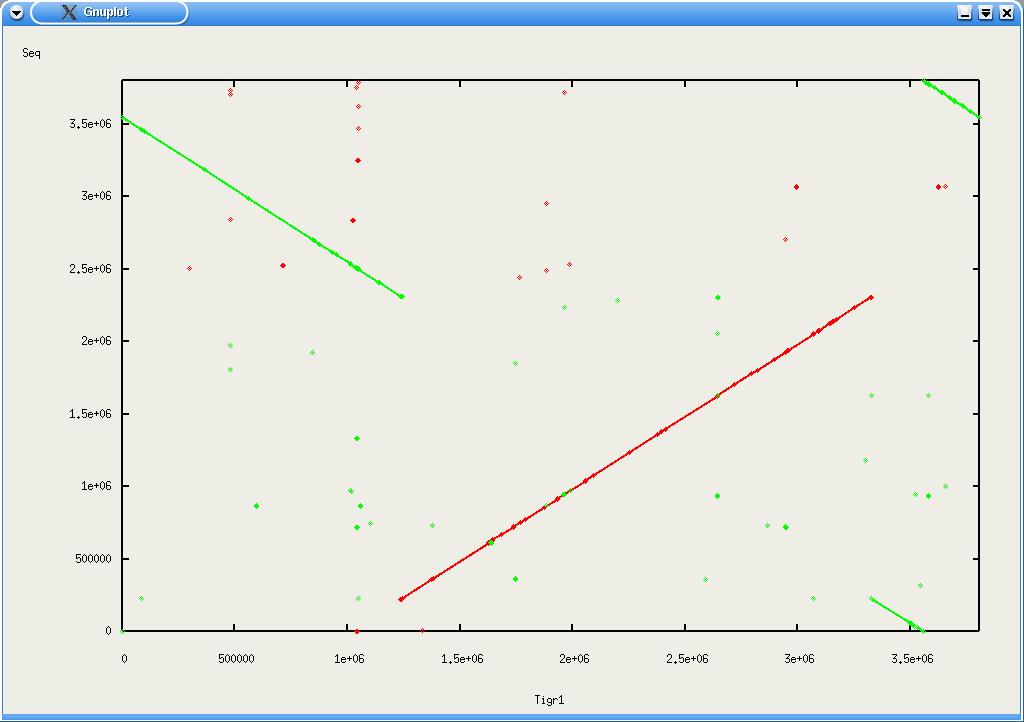

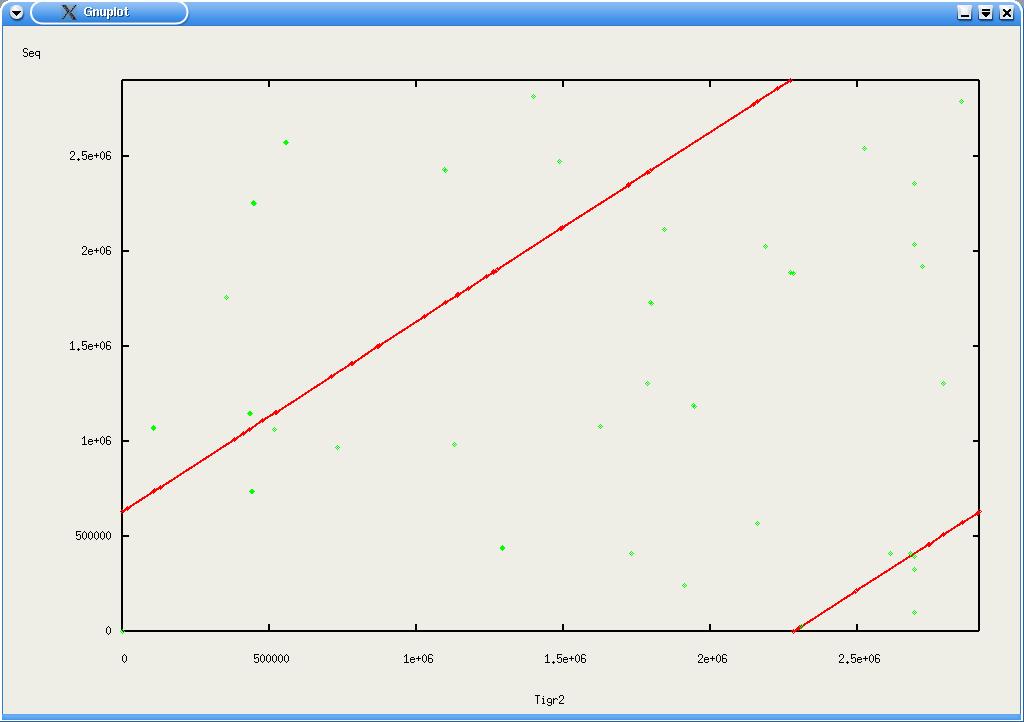


Genomic alignments of Chr 1 (left) and Chr 2 (right). The x-axis depicts the TIGR sequence, while the y-axis depicts the BI sequence.

It can be seen that a large-scale inversion of about 2 million bp has occurred in Chr 1. Using the TIGR sequence as a reference, the inversion stretches from position 12442442 (BTH_I1099) until 3328461 (BTH_I2895).

**2. Comparison at the CD level**

All 5645 CDs in the TIGR sequence were (3282 in chromosome 1 and 2363 in chromosome 2) compared to the BI sequence. We identified 4 CDs without clear matches in the BI sequence and which did not lie within or close to sequence gaps. Notably, BTH_I1485 and BTH_I1486 encode components of a Type-II oligopolysaccharide biosynthesis gene cluster. We experimentally confirmed the absence of these two genes in the BI sequence using PCR assays (P.T., data not shown).

**3. Comparison at the nucleotide level**

We identified a total of 218 sequence polymorphisms between the TIGR and BI sequence, covering both single nucleotide polymorphisms (SNPs) and small insertions/deletions (indels). 138 of these polymorphisms were on Chr 1, and 80 on Chr 2. 80 of these polymorphisms were predicted to cause alterations in protein sequence.To confirm these potential sequence differences, we selected 66 polymorphisms within 37 CDs for confirmatory resequencing (33 polymorphisms from 15 ORFs in chromosome 1 and also 33 polymorphisms from 22 ORFs from chromosome 2). The resequencing results only managed to positively confirm 1 polymorphism; another 9 polymorphisms appeared to be potentially genuine as well but they require another round of confirmation by resequencing. The remaining polymorphisms are either false (no such polymorphism: sequencing error) or cannot be confirmed due to poor sequence quality. These results are summarized in the following table.

| Type of polymorphism | | Number of ORF | |
| --- | --- | --- | --- |
|  |  | **Chromosome 1** | **Chromosome 2** |
| *Nucleotide substitution* | | *21* | *18* |
|  | Confirmed | 1 | 0 |
|  | Likely* | 7 | 2 |
|  | False | 9 | 4 |
|  | Poor data quality | 4 | 12 |
| Insertion | | *11* | *14* |
|  | Confirmed | 0 | 0 |
|  | Likely* | 0 | 0 |
|  | False | 11 | 13 |
|  | Poor data quality | 0 | 1 |
| *Deletion* | | *1* | *1* |
|  | Confirmed | 0 | 0 |
|  | Likely* | 0 | 0 |
|  | False | 1 | 1 |
|  | Poor data quality | 0 | 0 |
| *Total* | | *33* | *33* |

*: “Likely” denotes that the polymorphism is likely to be true and need more confirmation.

Genes Absent in ATCC700388 but Present in E264

Genes with Putative Protein Altering Polymorphisms between ATCC700388 and E264
