# Supplementary material for: Genomic patterns of pathogen evolution revealed by comparison of Burkholderia pseudomallei, the causative agent of melioidosis, to avirulent Burkholderia thailandensis
Source: BMC Microbiol. 2006 May 26;6:46. doi: 10.1186/1471-2180-6-46 (PMC1508146; doi:10.1186/1471-2180-6-46)
Supplement: Additional File 4 — Genome sequence of Bt E264 (Broad Institute). [file 1471-2180-6-46-S4.doc]

**Additional file 4: Genome sequence of Bt ATCC700388 (Broad Institute)**

*B. thailandensis* E264 (Singapore Variant) was sequenced to a total sequence coverage (fold) of 7x. The computer program ARACHNE was used to assemble the shotgun reads into contigs and scaffolds. The median contig length was 40 Kb (86 contigs in total). 3 scaffolds could be assembled from the contigs, comprising 3.4, 2.9, and 0.35 Mb respectively. A subsequent comparison of these scaffolds to the TIGR sequence allowed the 1^st^ and 3^rd^ scaffold to be collapsed into a single assembly (Chromosome 1). The amount of genome sequence contained in contigs (sum of contig lengths) was 6.66 Mb (Chr 1 – 3.78 Mb, Chr 2 – 2.88 Mb), compared with a total genome length (sum of scaffold lengths, including estimated gaps between contigs) of 6.7 Mb (Chr 1 – 3.80 Mb, Chr 2 – 2.9 Mb). Thus, approximately 99% of the *B. thailandensis* genome is contained in contigs. There are 43 sequence gaps, of which 15 gaps are larger than 500 bp (largest gap 9995 bp).
